# *Dmy* initiates masculinity by altering *Gsdf*/*Sox9a2*/*Rspo1* expression in medaka (*Oryzias latipes*)

**DOI:** 10.1038/srep19480

**Published:** 2016-01-25

**Authors:** Tapas Chakraborty, Lin Yan Zhou, Aparna Chaudhari, Taisen Iguchi, Y. Nagahama

**Affiliations:** 1 Laboratory of Reproductive Biology, National Institute for Basic Biology, Okazaki 444-8585, Japan; 2 SORST, Japan Science Technology Corporation, Kawaguchi, Saitama 332-0012, Japan; 3 South Ehime Fisheries Research Center, Institution for Collaborative Relations, Ehime University, Nishiura, 798-4206, Japan; 4 Key Laboratory of Aquatic Science of Chongqing, School of Life Science, Southwest University, Chongqing, 400715, China; 5 Central Institute of Fisheries Education, Mumbai, 400061, India; 6 Laboratory of Molecular Environmental Endocrinology, Okazaki Institute for Integrative Bioscience, National Institute for Basic Biology, Okazaki 444-8787, Japan; 7 South Ehime Fisheries Research Center, Institution for Collaborative Relations, Ehime University, Matsuyama 790-8577, Japan

## Abstract

Despite identification of several sex-determining genes in non-mammalian vertebrates, their detailed molecular cascades of sex determination/differentiation are not known. Here, we used a novel RNAi to characterise the molecular mechanism of *Dmy* (the sex-determining gene of medaka)-mediated masculinity in XY fish. *Dmy* knockdown (*Dmy*-KD) suppressed male pathway (*Gsdf, Sox9a2*, etc.) and favoured female cascade (*Rspo1*, etc.) in embryonic XY gonads, resulting in a fertile male-to-female sex-reversal. *Gsdf, Sox9a2*, and *Rspo1* directly interacted with *Dmy*, and co-injection of *Gsdf* and *Sox9a2* re-established masculinity in XY-*Dmy*-KD transgenics, insinuating that *Dmy* initiates masculinity by stimulating and suppressing *Gsdf*/*Sox9a2* and *Rspo1* expression, respectively. Gonadal expression of *Wt1a* starts prior to *Dmy* and didn’t change upon *Dmy*-KD. Furthermore, Wt1a stimulated the promoter activity of *Dmy*, suggesting Wt1a as a regulator of *Dmy*. These findings provide new insights into the role of vertebrate sex-determining genes associated with the molecular interplay between the male and female pathways.

Sex determination is the process that determines the sex of an organism. The signals that control this decision are usually categorized as genetic or environmental. In the genetic sex determination, four master sex-determining genes have been previously identified: *SRY/Sry*, most mammals including humans^1^; *Dmy/Dmrt1Y*, medaka^2^,^3^; *Dmrt1*, chicken^4^; *Dm-W*, *Xenopus laevis*^5^. More recently, novel autosomal sex-determining genes were reported in five teleost species: *Amhy*, *Odontesthes hatcheri*^6^; *Gsdf*, *Oryzias luzonensis*^7^; *Amhr2*, *Takifugu rubripes*^8^; *SdY*, *Oncorhynchus mykiss*^9^ and *Sox3*, *Oryzias dancena*^10^. In the past decade, it has become clear that although the upstream sex-determining signals are diverse, they often act through more ancient downstream regulatory hierarchies^11^.

Japanese medaka (*Oryzias latipes*) is one of the best-studied species among non-mammalian vertebrates with respect to sex determination and differentiation. Sex determination in medaka is strictly genetic, with a male heterogametic (XX/XY) system^2^. The testicular morphogenesis in medaka is clearly distinctive from the ovarian morphogenesis during the early stages of gonadal differentiation^12^. It is generally accepted that the number of germ cells, more specifically the proliferative mitosis and subsequent occurrence of meiosis in females and mitotic arrest in males by the day of hatching, are the first sign of morphological sex differentiation in medaka^12^. Thus, the day of hatching is an important time point on which the morphological sex differentiation becomes apparent in the medaka XY and XX gonads not only in the form of germ cell proliferation, but also meiosis. Furthermore, in the QurtE strain of Japanese medaka, genetic sex of the embryos can be identified by the presence or absence of leucophores on the head region from 2 days after fertilization (daf)^13^. Leucophores are present in the head region of XY embryos but not in XX embryos^13^.

In medaka, the Y chromosome harbours the master sex-determining gene (primary testis determinant), *Dmy/Dmrt1bY*^2^,^3^. A mutation due to a single nucleotide insertion in the exon 3 of *Dmy* ORF leads to a truncated Dmy, which gives rise to spontaneous sex reversed XY female medaka^2^,^14^. The overexpression of *Dmy* resulted in the induction of testis differentiation and subsequent male development in XX (genetically female) medaka. Moreover, gripNA induced knockdown of *Dmy* by our group^15^ showed signs of increased germ cell number and meiotic progression, as early as hatching day (indicators of feminization) in XY medaka. However, no studies have yet revealed the course of action of *Dmy* on somatic and germ cells that induces the male sexual differentiation. Although several male-associated genes like *Gsdf* (gonadal soma derived factor, a unique member of the TGF-β family)^16^, *Sox9a2* (also known as *Sox9b*, the orthologue of tetrapod *Sox9*)^17^,^18^, and *Dmrt1*^19^ have been suggested to be downstream of *Dmy*, no specific targets have been identified for *Dmy*. On the contrary, even though the key ovary determinant in medaka has not been defined, *R-spondins*, *estrogen receptor* β*2* (*ER*β*2*) and *Foxl2* have been shown to express specifically in XX gonads during sex determination and differentiation^20^,^21^,^22^,^23^.

Although several fish sex-determining genes have been identified by now, no elaborate knockdown study of these sex-determining genes is available. Therefore, it is of critical importance to study the effect of reduced *Dmy* expression on sex differentiation and maturation in medaka. Although ZFN/TALEN/CRISPER-based knockouts are gaining popularity, each one has their own advantages and disadvantages^24^. Antisense (AS) RNA has long been considered a promising technique for treating disease^25^. There are several mechanisms by which AS RNA may exert its effect, such as de-stabilization of endogenous mRNA^26^, production of small RNAs^27^ and activation of the siRNA/miRNA pathway^27^,^28^. Medaka and zebrafish (*Danio rario)* are excellent models for studying developmental processes, because of several advantages over higher vertebrates. For example, these egg-laying teleosts are small, have a rapid generation time and produce large number of offsprings. Also, their external fertilization and well-documented developmental processes makes gene manipulation studies much easier to perform. In the present study using medaka, we have developed an AS DNA vector-based approach to overcome the shortcomings of the effect of *Dmy* knockdown. Upon knockdown of *Dmy*, we were successful in generating a functional sex-reversed XY medaka with full fertilization potential. The gonadal sex reversal mechanism in medaka was associated with simultaneous suppression and induction of downstream male-associated and female-associated genes, respectively.

## Results

### Trans-generational antisense DNA-dependent knockdown in medaka

**Figure 1:**
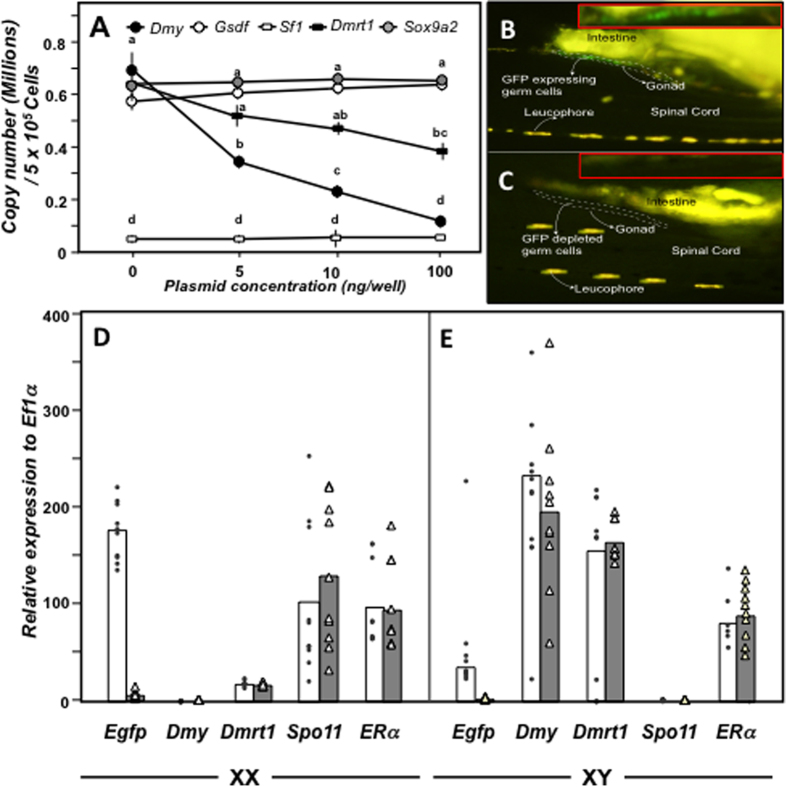
*In vitro* and *in vivo* validation of knockdown strategy. (**A**) *In vitro* validation. The specificity and off target effects of pmDMY-AS construct was validated *in vitro* using COS7 cells via co-transfection of pmDMY-AS construct and the ORF plasmids of either *Dmy* (specific, solid circles), *Dmrt1* (partially specific, solid squared), *Sox9a2* (non-specific, grey circles) *Gsdf* (non specific, open circles) or *Sf1* (non specific, open squared). The mean absolute copy numbers of respective gene per 5 ng of RNA are plotted in the graph along with SEM. The significance is indicated by a, b, c, in which different letters indicate the significant difference from other group at p < 0.05. (**B,C**) *In vivo* validation. *Olvas-eGFP* medaka were electroporated with pEGFP-AS plasmid (carries an antisense eGFP sequence) and continuous microscopic visualization (from 3days after fertilization (daf) to 5 days after hatching (dah)) of GFP expression in individual embryos was performed to ascertain the changes of GFP production in control (B, 6 daf) and pEGFP-KD (pEGFP-AS electroporated) groups (C, 6 daf). (**D,E**) Sharp fall in *Gfp* mRNA production was assessed through real-time PCR at 20 dah (days after hatching). Non-specific effects of pEGFP-AS construct were analysed by measuring the mRNA amount of *Dmy*, *Dmrt1*, *Spo11*, and *ERα*. Data are presented as both individual values (with dots and triangles) and mean of 10 individual (white and grey columns for control and knockdown groups, respectively) fish of each sex.

The present study used a 220 bp antisense DMY construct (neighbouring the DM domain) expressing short AS-RNA (Supplementary Fig. 1), which was designed and evaluated using E-RNAi (http://www.dkfz.de/signaling/e-rnai3//), to silence the *Dmy* gene in medaka. Gene specificity and possible off-target effects of the pmDMY-AS-construct on the downstream genes *Dmrt1, Sox9a2*, *Gsdf* and *Sf1* were studied in cell cultures co-transfected with pCMV-DMY, pCMV-DMRT1, pCMV-SOX9a2, pCMV-GSDF, and pCMV-SF1. Dose-dependent suppression of the *Dmy* (92% at 100 ng group) and *Dmrt1* (43.75% at 100 ng group) transcript were documented in this experiment. However, the expression of the other three genes (*Sox9a2, Gsdf* and *Sf1*) remained unaffected (Fig. 1A).

To confirm the knockdown effect of the AS technique *in vivo*, *Gfp* expression was knocked down in *olvas-eGFP* transgenic medaka (expresses GFP in the germ cells) one-two cell stage embryos^29^ by electroporation, using a pEGFP-AS plasmid (contains 320 bp antisense GFP sequence and expected to knockdown the GFP expression)^30^. Microscopic observations from 3 daf indicated a reduction of *Gfp* expression in gonads of electroporated embryos compared to negative controls (Fig. 1B,C). Real-time PCR using 20 dah (days after hatching) XX and XY fish demonstrated unchanged expression of other genes including *Dmy*, *Gsdf*, *Dmrt1, Spo11*, and *estrogen receptor* (*ER)* α (Fig. 1D,E). A group of pEGFP- knockdown (KD) fish was raised to adulthood to check whether the present method had some effect on gonad maintenance and secondary sexual character development in the long run. No side effect was observed with reference to growth and development of secondary sexual characteristics (Supplementary Table 1).

Our knockdown strategy is expected to transcribe AS RNA, which might be cleaved by endogenous RNA processing machineries (*e.g.*, dicer, RNaseH, etc.) and produce a pronounced knockdown effect. To elucidate the molecular mechanism of knockdown action, small-RNAs were isolated from sex-reversed F_1_ generation fish (mentioned later in detail) of *Dmy* knockdown (*Dmy*-KD) group. Northern blotting (using same 220 bp *Dmy* fragment as a probe) detected a strong signal at 60 base (b) and a weak signal in the 18–28 b region, only in *Dmy*-KD lane samples (Supplementary Fig. 2). Cloning and sequencing analysis (details in Supplementary Materials and Methods) of these bands revealed several fragments of both 60–70 b and 18–28 b were derived from the long *Dmy* AS sequence (Supplementary Fig. 2) and rest were randomly distributed throughout the *Dmy* mRNA sequence. We also sequenced the larger fragments (>100 bp size fragments) to find out any evidence for alternative splicing associated transcriptional rate inhibition. Both control-XY and *Dmy-*KD-XY fish had similar pattern of large RNA (>600 bp) fragmentation except the fact that, only *Dmy*-KD samples contained a trail for different size samples (most of them had portion of ORF/5UTR/3UTR but none showed any evidence for alternative splicing, especially for intronic sequences, joined or duplicated exons), and relative occurrence of full ORF was also significantly less (1/123.4 clones) than their control counterpart (1/4.7 clones). These observations suggest that the AS RNA-expressing construct mainly destabilizes the mRNA probably via small interfering RNA (siRNA)/micro RNA (miRNA) pathway to block the expression of *Dmy* (Supplementary Fig. 2D).

To test the above hypothesis about mode of knockdown, three representative shDNA (small hairpin DNA, each of 38 bp) were artificially synthesized using a Block-it™ pol II miR RNAi expression vector kit (Invitrogen, USA) following the manufacturer’s instructions. Precautions were taken while designing the shDNA constructs in order to make sure that all three shDNA fragments did not overlap each other and also belonged from the same targeted 220 bp *Dmy* region (used to prepare the pmDMY-AS construct). All these shDNA plasmids (pmCMV-DMYsh1, pmCMV-DMYsh2 and pmCMV-DMYsh3) were electroporated in one or two-cell medaka embryos in all possible combinations. pmDMY-AS and pEGFP-AS were used as positive and negative controls, respectively. Despite *Dmy* knockdown, the singularly injected pmCMV-DMYsh1, 2 or 3 specimens failed to show any significant phenotypic effect in terms of increased meiosis (Supplementary Table 2). The occurrence of mitosis and meiosis in male gonads increased approximately 2 fold upon double knockdown of pmCMV-DMYsh1 and 2. Triple knockdown using three short hairpin constructs, pmCMV-DMYsh1, 2 and 3, gave maximum knockdown comparable with pmDMY-KD (Supplementary Table 2). This suggests that, our pmDMY-AS construct reduces the mRNA stability and produces several small RNA, which in turn amplifies the knockdown effect.

### Functional sex reversal by *Dmy* knockdown

**Figure 2:**
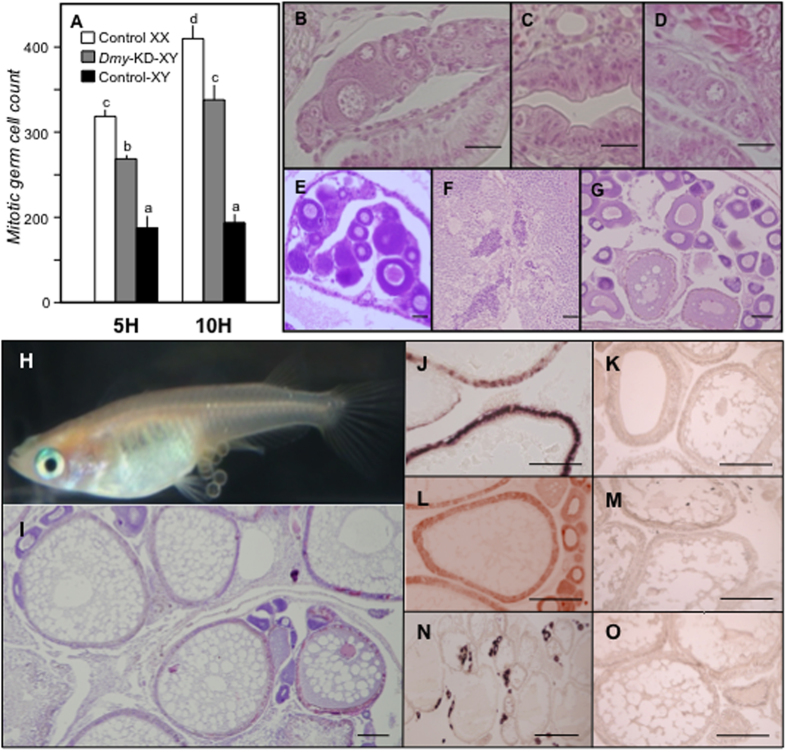
Effect of *Dmy* knockdown on gonad development. (**A**) The total number of mitotic germ cells counted (control XX, white; *Dmy*-KD XY, grey; control XY, black) at 5 dah (5H) and 10 dah (10H), was used as a marker for gonadal sex. Data are presented as mean of 10 individuals from both control and *Dmy*-KD groups. Error bars indicate SEM. Letters (a-c) above the bars indicate that these groups differ significantly (p < 0.05) from each other. (**B–G**) The candidate HE-stained sections of control XX females (**B,E**), control XY males (**C,F**), and *Dmy*-KD XY fish (**D,G**). Female-like gonad development is seen in *Dmy*-KD XY fish, which later forms a fully developed ovary. Note: B, C, and D represent gonads at 10 dah, while E, F, and G represent gonadal structure at 50 dah. (**H,I**) *Dmy* knockdown directed femininity was persistent during adulthood, which was confirmed by female-like external appearances (**H**) and gonad structure (**I**). The femininity was further confirmed by *ISH* with female (*Cyp19a1*, J; *Foxl2*, L; *Rspo1*, N), and male (*Dmy*, K; *Cyp11a*, M); *Cyp11b*, O) associated genes. (Scale bars, 20 μm.)

After checking the knockdown efficiency by real-time PCR analysis of several sex related genes including *Dmy* (discussed later), gonadal histology of *Dmy*-KD XY embryos was performed to assess the changes at the phenotypic level caused by *Dmy* suppression. Cells in early stages of meiosis were observed in XY gonads even at 0 dah and gradually increased at 5 and 10 dah (Fig. 2A–D). To observe the duration of the silencing effect, the electroporated XY fish were stained with haematoxylin and eosin (HE) at 50 dah (Fig. 2E–G). 50% of the fish were found to have fully grown ovaries containing variable numbers of oocytes, while 20% had testicular tissue in the gonad (Supplementary Table 3).

Further, genotyping was carried out and the sex-reversed *Dmy*-KD XY F_0_ females (Fig. 2H,I) were raised to maturity and five individuals were mated with normal males to test their mating and breeding ability. Although all the five F_0_ fish gave rise to viable progeny, complete AS construct was integrated into the genome of two specimens (here after considered as founder fish), as evidenced by the amplification of the transgene sequence (using a vector specific forward primer and a *Dmy* fragment specific reverse primer) from genomic DNA isolated from caudal fin clips (data not shown). We did not find any significant differences between progenies from these two founder fish (Supplementary Table 4). Hence, the progenies generated from these two F_0_ phenotypic females were used for further analysis.

In two specimens of F_1_ generation fish (out of ten checked by HE staining), meiotic cells were detected at 10 dah stage, which indicated that the antisense *Dmy* transgene (CMV-DmyAS) had passed to the next generation. However, the low level of gene transmission might be associated with the crossing method, where control XY fish were used as male partner. The remaining F_1_ progeny were raised to adulthood till the development of secondary sexual characters. Three sex reversed XY females (confirmed by the presence of male-specific leucophore (Y linked character) and *Dmy*-transgene amplification) from the F_1_ group were similarly analysed for mating and breeding behaviour. All tested fish were fully fertile and further mated to produce viable progeny (Supplementary Table 3). Similar analyses were performed in each generation before mating the animals for progeny production. The fecundity (egg number) of the *Dmy*-KD XY females was also quite similar to that of control XX females in different generations (from F_2_-F_5_, Supplementary table 5). We used the F_3_ fish to generate pmDMY-KD line in which the *Dmy* mRNA expression was very low (measured by real-time PCR in embryonic gonads) from early development period.

### Down-regulation of male-associated genes by *Dmy* knockdown

**Figure 3:**
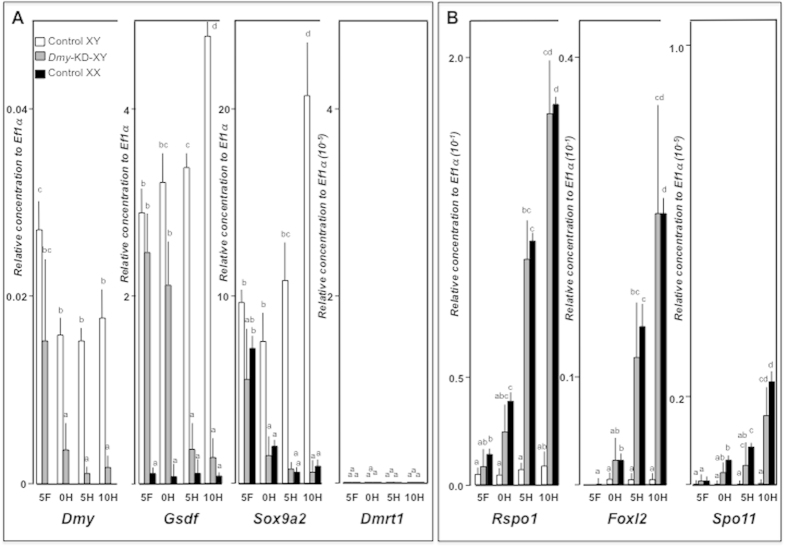
*Dmy*-knockdown induced transcriptional alterations of male and female associated genes. (**A,B**) Ontogenic changes in the expression of male (*Dmy*, *Gsdf*, *Sox9a2*, and *Dmrt1*) (**A**) and female (*Rspo1*, *Foxl2*, and *Spo11*) (**B**) associated genes from 5 daf (5F) to 10 dah (10H) were analysed by real-time PCR. Data were normalised with *Ef1a* expression. Each column in the graph represents mean ± SEM of 10 individuals of control XY (white columns) and *Dmy*-knockdown XY (grey columns) fish. Letters above the bars indicate that these groups differ significantly (p < 0.05) from each other.

To determine the effect of *Dmy* knockdown, real-time PCR was performed using samples from 5 daf (days after fertilization) to adulthood. As expected, at early stages, transient *Dmy* knockdown reduced the *Dmy* expression by 66–92%, even from 5 daf (p < 0.05). At 5 daf, there was slight reduction in the expression of other male-associated genes like *Gsdf* and *Sox9a2*. At 0 dah, the reduction rate of *Sox9a2* expression was much larger than *Gsdf*. At 5 and 10 dah, both *Gsdf* and *Sox9a2*, resulted in approximately 80–90% reduction compared to controls (p < 0.05) (Fig. 3A). Similar trend was also observed in the adults. Until 10 dah, there was no *Dmrt1* expression in both the knockdown and control groups. However, in the knockdown group, *Dmrt1* expression was drastically reduced in adulthood (p < 0.05) (Supplementary Figs 3, 4). All these results were further substantiated using *in situ* hybridisation (*ISH*). Among the fully-grown *Dmy*-KD XY fish, only the partially sex reversed fish showed low expression (p < 0.05) of *Dmy*, *Gsdf* and the spermatogonial markers (*Cyp11a* and *Cyp11b*) (Fig. 2K,M,O & S3). On the other hand, the fully developed pmDMY-KD XY ovary did not show any sign of spermatogonial marker gene expression but had female-like expression of *Gsdf* and other male dominated genes (p < 0.05) (Supplementary Fig. 3).

### Up-regulation of female-associated genes by *Dmy* knockdown

To determine the effect of *Dmy* knockdown on the expression of female-associated genes, real-time PCR was carried out using *Dmy*-KD fish. *Rspo1* showed increased expression at 5 daf in knockdown fish, which became more evident at later stages in comparison to control-XY groups (p < 0.05). However, *Foxl2* could only be differentiated from 0 dah onwards. Interestingly, when compared to controls, all these female-specific genes were markedly upregulated from 5 dah (p < 0.05) (Fig. 3B). In adults, except for four fish, all showed a substantial increase in the expression of female-associated genes compared to their respective controls (Supplementary Fig. 3). *Dmy-*KD-induced feminization was further confirmed by *ISH* (Fig. 2J,L,N). All the sex-reversed *Dmy*-KD XY fish (both partial and complete) showed elevated *Spo11*, *Foxl2*, *Rspo1*, and *Cyp19a1 (ovarian aromatase)* expression (Supplementary Fig. 3), while unchanged fish had male-alike or no expression of these genes.

### Role of Wt1a in the regulation of *Dmy* expression

**Figure 4:**
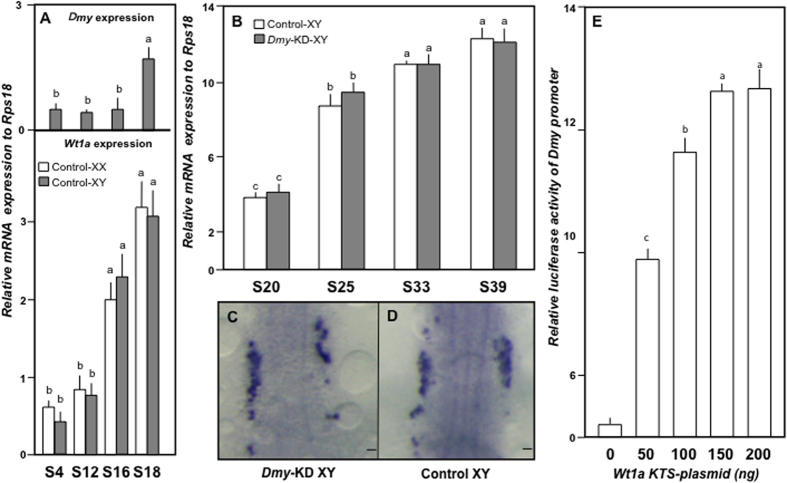
Wt1, an upstream candidate for *Dmy* regulation. (**A**) *Dmy* and *Wt1a* expression were analysed using several early stage samples (S4-S18) to assess the precise start point of zygotic expression (XX, white columns and XY, grey columns). (**B**) Differences in *Wt1a* expression between control (white columns) and *Dmy*-KD (grey columns) XY embryos during gonadal differentiation (S20-S39) were also analysed by real-time PCR. (**C,D**) *Wt1a* specific whole mount *ISH* of *Dmy*-KD XY (**C**) and control XY (**D**) embryos (stage 22) showed no virtual difference. (**E**) Luciferase assays using HEK293 cells showed a dose dependent activation of *Dmy* promoter by *Wt1a* KTS (−). Graphical data are presented as an average of three independent experiments, each containing triplicates of individual sampling group. Error bars represent SEM. Letters (a–c) above the bars indicate that these groups differ significantly from each other at p < 0.05.

In mammals, Wt1 is known to be an important regulator of *Sry*. Duplicated copies of *Wt1* (*Wt1a* and *Wt1b*) have been reported in teleosts, including medaka^31^. In order to understand the isoform-specific effects of Wt1 on *Dmy* regulation, we characterised the expression patterns of *Dmy*, *Wt1a* and *Wt1b* during gonadal differentiation in both XY and XX fish, including the F_3_ *Dmy* KD transgenics (pmDMY-KD XY). *Wt1a* starts its zygotic expression from stage 12 (10 hours after fertilization, haf) (Fig. 4A), while *Wt1b* sets in from stage 25 (2 daf) (data not shown). Interestingly, *Dmy* initiated its zygotic expression from stage 18 (1 daf) (Fig. 4A), which was much later than *Wt1a* expression but earlier than *Wt1b* expression. This gave rise to a possibility of *Wt1a* as a candidate regulator of *Dmy* in medaka. Thereafter, we examined the effect of *Dmy* knockdown on *Wt1a* expression. Our real-time PCR and whole-mount *ISH* data showed identical expression between pmDMY-KD XY and control XY embryos at different stages (Fig. 4B–D).

In the subsequent experiment, we found a highly conserved Wt1-binding site (GAGGGGGGAG) in the *Dmy* promoter region^32^. Reportedly, in mammals, *Wt1* KTS (+) alternatively spliced isoform regulates the SRY transcription in a dose responsive manner^32^. To determine the role of Wt1(s) in the regulation of *Dmy* expression, a luciferase assay was performed using four Wt1-ORF plasmids (*Wt1a* KTS (+/−) and *Wt1b* KTS (+/−)) and *Dmy* promoter construct. To our surprise, only the *Wt1a* KTS (−) isoform showed dose-dependent activation of *Dmy* promoter-driven luciferase production (Fig. 4E).

### Rescue of *Dmy* knockdown effects by co-overexpression of *Gsdf* and *Sox9a2*

**Figure 5:**
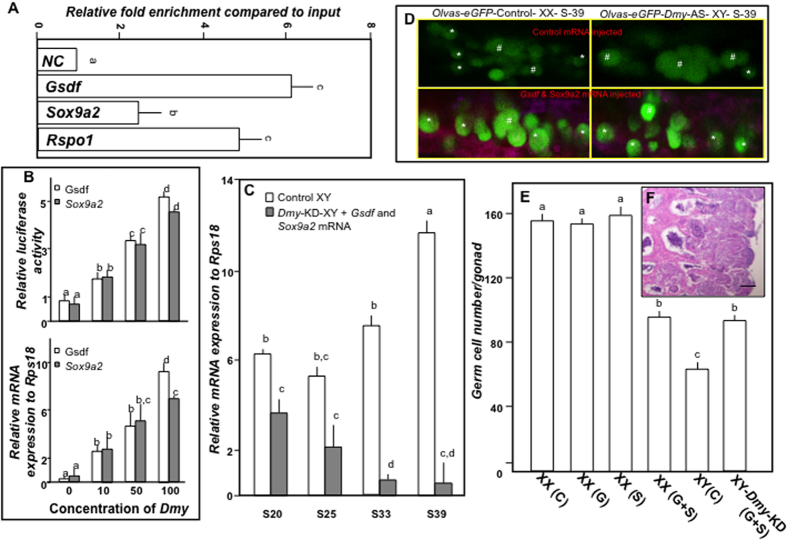
*Gsdf* and *Sox9a2* are potential downstream targets of *Dmy*. (**A**) *In vivo* chromatin immunoprecipitation analysis of candidate *Dmy* targets. Relative expression to control input was calculated using real-time PCR and plotted on the graph. Data are presented as means ± SEM of 4 separate experiments and significances (p < 0.05) are denoted by different letters. (**B**) Effects of *Dmy* overexpression on *Gsdf* and *Sox9a2* promoter activity (upper panel) and mRNA transcription (lower panel) were ascertained. (**C**) Differences in *Dmy* expression between control (white columns) and *olvas-eGFP-Dmy-*KD co-injected with *Gsdf-cherry* and *Sox9a2-cyan* (grey columns) XY embryos during gonadal differentiation (S20-S39) were assessed using real-time PCR. (**D**) Representative images showing the germ cell distribution in *Olvas*-*eGFP*-control XX, *Olvas*-*eGFP*-*Dmy*-KD-XY embryos at S39 after control (upper panel) and *Gsdf/Sox9a2* (lower panel) mRNA injection. Isolated and cluster germ cells are marked with ‘*’ and ‘#’, respectively. Note: Clustered germ cells are representative of female type gonadal development (N = 9/ group). (**E**) The total number of germ cells was plotted (Y-axis) against different injected groups (X-axis). Data are presented as means of 10 individuals (error bars represent SEM). Different letters (a–d) indicate significant differences from other groups at p < 0.05. Note: C, G, S, and G + S represents PBS, *Gsdf-cherry*, *Sox9a2-cyan*, and *Gsdf-cherry*/*Sox9a2-cyan* mRNA injected embryos, respectively. (**F**) The gonadal sexuality of *olvas*-*eGFP*-*Dmy*-KD embryos co-overexpressed with *Gsdf*-*Sox9a2* (N = 7) was assessed at maturity. (Scale bar, 20 μm).

To find out the direct downstream target of *Dmy*, we performed chromatin immuno-precipitation (ChIP) using *Dmy* (tagged with eGFP) mRNA overexpressed 6 daf medaka embryos. Since we observed differential but significant *Dmy* knockdown dependent down-regulation of *Gsdf* and *Sox9a2* and up-regulation of *Rspo1*, the potential downstream targets for *Dmy*, these genes were chosen as candidates for ChIP analysis. The primers were selected based on the availability of *Dmy* binding site (wacawtgtwk)^33^ in both upstream (−20 kb) and downstream (+10 kb) region of candidate genes. We found strong *Gsdf* and *Rspo1* (4–6 and 3.5–5 fold, respectively, depending on the area tested), but weak *Sox9a2* (1.59–2.87 fold) enrichment from 6 daf *Dmy-GFP* mRNA-injected embryonic samples (details in Supplementary Materials and Methods), determined by ChIP, using GFP antibody (Fig. 5A, Supplementary Fig. 5). Recently we published that, *Rspo1* promoter activity was inversely regulated by *Dmy*^34^. To get a better idea about *Dmy* association in *Gsdf* and *Sox9a2* regulation, we performed *in vitro* promoter assay of both *Gsdf* and *Sox9a2* promoters using OL32 cell line. Although the Ol32 cells possess slight *Dmy*, *Gsdf* and *Sox9a2* transcription of its own, the luciferase activity of both promoters and transcription of *Gsdf* and *Sox9a2* showed significant increment after *Dmy* overexpression, in a dose dependent manner (Fig. 5B). Owing to the transcriptional history of medaka and our data, it is highly likely that *Dmy* directly promotes the transcriptions of both *Gsdf* and *Sox9a2*, and simultaneously suppresses *Rspo1* to maintain the masculinity. If these assumptions are correct, then *Gsdf* and *Sox9a2* will alone or together be sufficient to rescue the *Dmy* knockdown effect. To prove that, *Gsdf* (tagged with mCherry) and *Sox9a2* (tagged with cyan) mRNA were singularly injected or co-injected into both *olvas-eGFP* XX and *olvas-eGFP Dmy*-KD XY embryos (F_3_ generation). Although singular injections failed to completely suppress meiosis and proliferative mitosis, co-injection re-established the male phenotype in the XY gonad, leading to the complete formation of testis (Fig. 5C–F, Supplementary Fig. 6). However, the amount of *Dmy* remained substantially low (Fig. 5C). Interestingly, the *Gsdf* and *Sox9a2* co-injected control *olvas-eGFP* XX embryos were also redirected towards male type gonadal development, i.e. restriction in germ cell proliferation (Fig. 5D,E).

## Discussion

In the present study, we developed a unique, relatively stable, cost-effective and gene-specific RNAi delivery system, capable of producing multiple small RNA against a specific gene, *Dmy*, in medaka. A DNA vector-based approach was used, owing to its many advantages over other approaches, like better stability, prolonged retention, ease of transmission and cost effectiveness^35^,^36^. The long AS molecule produced by this vector was useful in inhibiting the gene expression by promoting degradation of double-stranded RNA, perhaps by Dicer and miRNA/siRNA mechanism^37^. Availability of several kinds of unique small RNA helped us to hypothesize that AS-RNA were processed by the cell machinery to generate several small RNA (60–70 nucleotides) and siRNA (18–28 nucleotides) molecules^27^,^28^ and increase the silencing efficiency. Moreover, due to continuous transcription, long AS-RNAs were expected to be constitutively transcribed *in vivo*, thus blocking the translation of messenger RNA before being degraded^38^. The hypothesis was further confirmed by specific knockdown of *Gfp* expression in *olvas-eGFP* transgenic medaka. The specificity of *Gfp* knockdown was undoubtedly confirmed by the microscopic reduction of *Gfp* expression in the gonads. However, unlike shrimp^39^, in the present study, we did not observe any suppression in growth and reproduction of knockdown fish.

This newly developed knockdown technology was used to investigate the function of the zygotic gene product, *Dmy*, in medaka throughout its life span. Upon knockdown of *Dmy*, we were successful in generating a functional sex-reversed XY medaka with full fertilization potential. The marked suppression of *Dmy*, even at 5 daf, suggests the efficacy of knockdown. Being the master sex determination regulator in medaka, it is very important to know the detailed role of *Dmy* during gonadal determination, development and maturation. In medaka, early appearance of progressive mitosis and meiosis is the first sign of morphological sex determination in females. However, in males, the occurrence of *Dmy* arrests mitosis and drives the male sex determination. In the present study, we could also observe meiosis in *Dmy*-KD XY gonads even at 0 dah, confirming the previous findings which showed a surge in *Scp3* production and mitotic proliferation in XY DMY-gripNA and morpholino knockdown medaka^15^,^40^.

Knockdown of *Dmy* also induced marked reduction of two major male-associated autosomal genes, *Gsdf* and *Sox9a2*, during early gonadal sex differentiation in XY fish. We have previously shown that a rise in *Gsdf* expression is seen specifically in genetic males around 6 daf, almost the same time at which *Dmy* expression takes the peak^16^. Importantly, *Dmy* and *Gsdf* were found to be co-localized in the same somatic cells in the XY gonads^16^. Although there was very little reduction in *Gsdf* expression at 5 daf after knockdown of *Dmy*, a marked reduction was evident at 5 and 10 dah. Similarly, ChIP and promoter analysis also depicted direct regulation between *Gsdf* and *Dmy.* This suggests that male oriented gonadal fate determination was initiated by *Dmy* expression and further progressed by *Dmy* and *Gsdf* in a co-operative manner^16^. This also supports our previous hypothesis that *Gsdf* is a potential target of *Dmy* in male gonad development in XY Japanese medaka (*O. latipes*)^16^. Intriguingly, recent findings indicate that *Gsdf* is the master sex-determining gene in the Hainan medaka, *Oryzias curvinotus*, a closely related species of medaka^7^. Although *Dmy* is not present in *O. luzonensis*, the spatial and temporal expression pattern of *Gsdf* is closely correlated to *Dmy* in medaka^16^ and the expression patterns of more downstream genes in testicular differentiation, such as *Sox9a2* and *Dmrt1*, are similar in both medaka species^7^,^23^,^41^. Therefore, *O. latipes* and *O. luzonensis* share a common sex differentiation pathway downstream of *Gsdf* and, if high *Gsdf* expression is achieved during sex differentiation, then the XX embryo develops as a male without *Dmy*^7^.

In a number of higher vertebrates, *Sox9* has been shown to play a major conserved role in testis determination. In mice, from 10.5 dpc, the Y-linked sex-determining gene *Sry* is expressed in XY genital ridges and initiates *Sox9* expression and testis differentiation^42^. Knocking-down of *Sry* expression resulted in significant decrease in the *Sox9* expression and gonadal feminization of mouse embryos^43^. Similarly, in chicken, *Sox9* expression was significantly reduced in *Dmrt1* knockdown genetically male (ZZ) gonads relative to controls^4^. In medaka, *Sox9a2* (a duplicated homologue of mammalian *Sox9*) is initially expressed in somatic cells of both sexes, but upregulated in testicular somatic cells and down regulated in the XX gonads, at 10 to 30 dah^17^. Contrastingly, Nakamura *et al.*^18^, using both transgenic and chimeric *Sox9a2* (*Sox9b*) medaka mutants, reported that *Sox9a2* is required for germ cell proliferation and survival, but not for testis determination^17^. However, we previously depicted a significant elevation of *Sox9a2* at 10 dah XY fish, suggesting its role in testis development^17^,^18^. In the present study, upon *Dmy* knockdown, there was a marked reduction of *Sox9a2* expression even from 0 dah onward. These results suggest that *Sox9a2* is downstream of *Dmy* and possesses some role in the early stages of testis differentiation. Although significantly lower, the precipitation of *Sox9a2* DNA in *Dmy*-ChIP experiment suggests a direct link between *Sox9a2* and *Dmy*, which is substantiated by our promoter analysis data. Further in-depth analysis is required to clarify the role of *Dmy*-*Sox9a2* interaction in testis differentiation and development in medaka.

As stated above, the expression of two major male-associated genes, *Gsdf* and *Sox9a2*, were markedly reduced in *Dmy*-KD XY fish. In the present study, the availability of F_3_ *Dmy*-KD transgenics gave us the option to study the downstream targets of *Dmy* by overexpressing candidate down-stream genes such as *Gsdf* and *Sox9a2*. Our results showed that although individual overexpression of either *Gsdf* or *Sox9a2* induced partial recovery of male phenotype, co-overexpression of these two genes could fully re-establish the male pathway, producing fertile sperm in *Dmy*-KD fish. These results, along with the ChIP data, suggest some parallel and essential role of these two genes in testicular differentiation and development. Since *Gsdf* and *Sox9a2* are expressed in the same somatic cells in medaka XY gonads, it is quite possible that, like mammalian *Sry*/*Sox9*-*Fgf9* regulation^11^, *Dmy* could simultaneously induce both *Gsdf* and *Sox9a2*, which in turn influences each other to maintain masculinity. However, no such evidence has been reported till date in medaka.

Unlike *Gsdf* and *Sox9a2*, in XY medaka gonad, *Dmrt1* expression is initiated only after the formation of testis (20–30 dah). Recently, we found that *Dmrt1* mutant XY medaka develops gonad that initially appear to be males but later becomes fertile XY females^44^. These results indicate that *Dmrt1* is essential to maintain testicular identity after *Dmy*-triggered male differentiation pathway. In the present study, we found a drastic reduction of *Dmrt1* expression in sex-reversed XY adults, further confirming the significance of *Dmrt1* in maintaining testicular differentiation and development.

In this study, *Dmy* knockdown caused complete suppression of male-associated genes in XY gonads, and generated sex-reversed XY fertile females, which thereby highlights the essentiality of female/ovarian-associated genes in *Dmy*-reduced condition. Although the key ovarian determinant has not yet been identified, we now know that a number of female-associated factors, such as canonical Wnt signaling through *Wnt4*, *Rspo1* and β*-catenin*, and *Foxl2*, are critical for ovarian differentiation in vertebrates^45^,^46^. Recently, much attention has been paid to the role of Rspo-activated signaling pathway in early sex determination and differentiation. *Rspo1* displays a conserved, female-specific increase in expression in several vertebrate species^33^,^47^. In medaka, *Rspo1* is predominantly expressed in XX gonads during sex differentiation, immediately preceding the first signs of ovarian differentiation, suggesting that *Rspo1* is a key player in medaka ovarian differentiation^20^. In the present study, we observed significant elevation in *Rspo1* expression at 0 dah in *Dmy*-KD XY fish. It is important to note that at this stage *Dmy* expression is already markedly down regulated, but *Gsdf* expression is still elevated. These findings suggest that *Dmy* expression, but not *Gsdf* expression, in males can block the *Rspo1* signalling. It has been proposed that the fate of the developing mammalian gonad involves a tug of war between the Sry/Sox9-induced Fgf9 signalling (male promoting) versus the Wnt signalling (female promoting)^46^. In this connection, it is important to note that, *Rspo1* directly interacts with *Dmy* (as evidenced by ChIP) and *Rspo1* expression is localized to somatic cells surrounding germ cells as well as germ cells during early developmental stages^20^. Further studies are required to determine whether a similar antagonistic system (the Dmy/Gsdf signalling in males versus the Rspo1 signalling in females) also applies in medaka.

*Foxl2* is also known to be involved in ovarian differentiation in a number of vertebrate species^45^. More recently, *FOXL2* was identified as a bona fide female sex-determining gene in goat^48^. In medaka, *Foxl2* is expressed only in the female gonads during gonadal differentiation, which starts around the day of hatching. In *Dmy*-KD XY fish, *Foxl2* expression was markedly upregulated at 5 dah, a time that coincides well with the marked fall in *Gsdf* expression in the *Dmy*-KD fish. These findings may suggest that there is a counter interaction between *Gsdf* and *Foxl2*, but this remains to be elucidated.

The important role of the Rspo1 and Foxl2 appears to be conserved from fish to mammals. In medaka, *Rspo1* expression precedes *Foxl2* by 2–3 days. It is important to determine how these two genes interact to coordinate ovarian determination and differentiation. We recently showed that in medaka, the expression profiles of *Rspo1*–*3* in gonads were markedly upregulated during a short period of oestrogenic exposure in 0 dah XY fish^20^, and the expression of *ER*β*2*, but neither *ER*α nor *ER*β*1*, exhibited marked increase in XX embryos between 6 and 8 daf with a peak at 7 daf^21^. It was also shown that *Foxl2* upregulated *Cyp19a1* gene transcription in a female-specific manner in other fish^22^. Thus, it is possible that oestrogen may be an intervening factor between Rspo1 and Foxl2 during early ovarian differentiation in medaka.

Although knockdown of *Dmy* gave rise to functional XY sex reversal, the exact molecular mechanisms involved in the regulation of *Dmy* expression in fish sex determination, is still vaguely understood. In mammals, a number of genes have been implicated as potential regulators of *Sry* expression, such as *Gata2*, *Gata4*, *M33*, and *Wt1*^42^. The present study focuses on the role of one of these genes, *Wt1*, in the regulation of *Dmy* during early testicular differentiation. In medaka, *Wt1a* is one of the primary genes expressed in somatic cells (precursor cells for *Dmy* expression) of bipotential gonads and first appears at Stage 12, preceding the zygotic expression of *Dmy*^31^. This highlights the importance of *Wt1a* in the regulation of *Dmy*. Unlike male and female-associated genes, *Wt1a* expression showed no change between control and *Dmy*-KD XY fish, suggesting that *Wt1a* is upstream of *Dmy*. Confirming this hypothesis, our results showed that *Wt1a* enhances the promoter activity of *Dmy* in a dose-dependent manner. Similarly, in mammals, the *Wt1a* KTS (−) and KTS (+) isoforms acts on transcription and RNA stability of *Sry*, respectively^49^,^50^. Taken together, these data suggests a conserved role of *Wt1a* in regulating the two sex-determining genes, *Sry* and *Dmy*. It is of interest to determine whether *Wt1* can also regulate the expression of chicken *Dmrt1*^4^, *Xenopus laevis Dm-W*^5^ and other novel fish specific sex determining genes, i.e. *Gsdf*, *Amh, Amhr2, SdY, and Sox3*^6^,^7^,^8^,^9^.

In conclusion, the new gene-specific transgenic RNAi technology developed for medaka has made it possible for the first time to investigate the function of *Dmy* in XY fish throughout its life span. A distinct shift in gene expression patterns from *Gsdf*/*Sox9a2* to *Rspo1*/*Foxl2* occurs in the germ cells surrounding somatic cells of *Dmy*-KD XY embryos, leading to a functional male to female sex reversal. Further studies, using this *Dmy* knockdown line, along with other related transgenics/mutants, are required to understand the 1) exact roles of *Gsdf* and *Sox9a2* in testicular differentiation and development and 2) life long tug of war between male/female associated genes in determining or maintaining the gonadal sex. The probable limitations of this method concerning the sterility of homozygous and heterozygous animals and associated hurdles of transgenic line maintenance need to be critically investigated, using fertility-associated genes.

## Materials and Methods

### Ethics statement

The studies were carried out in accordance with the Institutional Ethics Committee of National Institute for Basic Biology, Okazaki, Japan, strictly adhering to the guidelines set for the usage of animals by this committee. All *in vivo* experiments and fish maintenance were conducted following protocols and procedures approved by the Institutional Animal Care and Use committee at National Institute for Basic Biology, Japan. All surgery was performed under Tricaine-S anesthesia, and all efforts were made to minimize suffering.

### Experimental procedures

Detailed description of plasmid constructs, gene knockdown in Cos7 cells, experimental animals, knockdown of *Dmy* expression in medaka embryos, sampling, histology and *in situ* hybridisation, quantification of changes in gene expression by real-time PCR, assessment of the trans-generational knockdown effect, characterisation of small RNAs, promoter analysis of *Dmy*, Immunoprecipitation analysis and rescue of *Dmy* knockdown effect can be found in Supplementary Materials and Methods. Primer sequences for quantitative PCR and *ISH* probes are provided in Supplementary table 6.

## Additional Information

**How to cite this article**: Chakraborty, T. *et al.* *Dmy* initiates masculinity by altering *Gsdf/Sox9a2/Rspo1* expression in medaka (*Oryzias latipes*). *Sci. Rep.* **6**, 19480; doi: 10.1038/srep19480 (2016).

## Supplementary Material

Supplementary Information

## References

[b1] Sinclair A. H. *et al.* A gene from the human sex determining region encodes a protein with homology to a conserver DNA-binding motif. Nature 346(6281), 240–4 (1990). 1695712 10.1038/346240a0

[b2] Matsuda M. *et al.* *DMY* is a Y-specific DM-domain gene required for male development in the medaka fish. Nature 417(6888), 559–63 (2002). 12037570 10.1038/nature751

[b3] Nanda I. *et al.* A duplicated copy of *DMRT1* in the sex-determining region of the Y chromosome of the medaka, *Oryzias latipes*. Proc. Natl. Acad. Sci. USA 99(18), 11778–83 (2002). 12193652 10.1073/pnas.182314699 PMC129345

[b4] Smith C. A. *et al.* The avian Z-linked gene *DMRT1* is required for male sex determination in the chicken. Nature 461(7261), 267–71 (2009). 19710650 10.1038/nature08298

[b5] Yoshimoto S. *et al.* A W-linked DM-domain gene, DM-W, participates in primary ovary development in *Xenopus leavis*. Proc. Natl. Acad. Sci. USA 105(7), 2469–74 (2008). 18268317 10.1073/pnas.0712244105 PMC2268160

[b6] Hattori R. S. *et al.* A Y-linked anti-Müllerian hormone duplication takes over a critical role in sex determination. Proc. Natl. Acad. Sci. USA, 109(8), 2955–9 (2013). 10.1073/pnas.1018392109 PMC3286941 22323585

[b7] Myosho T. *et al.* Tracing the emergence of a novel sex-determining gene in medaka, Oryzias luzonensis. Genetics 191(1), 163–70 (2012). 22367037 10.1534/genetics.111.137497 PMC3338257

[b8] Kamiya T. *et al.* A Trans-species missense SNP in *Amhr2* is associated with sex determination in the Tiger Pufferfish, *Takufugu rubripes* (Fugu). PLoS Genet. 8(7), e1002798 (2012). 22807687 10.1371/journal.pgen.1002798 PMC3395601

[b9] Yano A. *et al.* An immune-related gene evolved into the master sex determining gene in rainbow trout *Oncorhynchus mykiss*. Curr. Biol. 22(15), 1423–8 (2012). 22727696 10.1016/j.cub.2012.05.045

[b10] Takehana Y. *et al.* Co-option of *Sox3* as the male-determining factor on the Y chromosome in the fish Oryzias dancena. Nat. Comm. 5, 4157 (2014). 10.1038/ncomms5157 24948391

[b11] Clinton K. M. & Zarkower D. Sex and the singular DM domain: insights into sexual regulation, evolution and plasticity. Nat. Rev. Gen. 13(3), 163–74 (2012). 10.1038/nrg3161 PMC3595575 22310892

[b12] Kobayashi T. *et al.* Two DM domain genes, *DMY* and *DMRT1*, involved in testicular differentiation and development in the medaka, *Oryzias latipes*. Dev. Dyn. 231(3), 1518–1526 (2004). 10.1002/dvdy.20158 15376325

[b13] Wada H. *et al.* Sex- Linked inheritance of the lf locus in the medaka fish (*Oryzias latipes*). Zool. Sci. 15(1), 123–126 (1998). 18429663 10.2108/zsj.15.123

[b14] Okate H. *et al.* The medaka sex-determining gene *DMY* acquire a novel temporal expression pattern after duplication of *DMRT1*. Genesis 46(12), 719–23 (2008). 18821592 10.1002/dvg.20431

[b15] Paul-Prasanth B. *et al.* Knock-down of *DMY* initiates female pathway in the genetic male medaka, *Oryzias latipes*. Biochem. Biophys. Res. Commun. 351(4), 815–9.(2006). 17092483 10.1016/j.bbrc.2006.10.095

[b16] Shibata Y. *et al.* Expression of gonadal soma derived factor (GSDF) is spatially and temporally correlated with early testicular differentiation in medaka. Gene Expr. Patterns 10(6), 283–9 (2010). 20601164 10.1016/j.gep.2010.06.005

[b17] Nakamoto M. *et al.* Testicular type *Sox9* is not involved in sex determination but might be in the development of testicular structure in medaka, *Oryzias latipes*. Biochem. Biophys. Res. Commun. 333(3), 729–36 (2005). 15963466 10.1016/j.bbrc.2005.05.158

[b18] Nakamura S. *et al.* Analysis of medaka *sox9* orthologue reveals a conserved role in germ cell maintenance. PLoS One 7(1), e29982 (2012). 22253846 10.1371/journal.pone.0029982 PMC3257256

[b19] Kobayashi T. *et al.* Two DM domain genes, *DMY* and *DMRT1*, involved in testicular differentiation and development in the medaka, *Oryzias latipes*. Dev. Dyn. 231(3), 1518–26 (2004). 10.1002/dvdy.20158 15376325

[b20] Zhou L. Y. *et al.* R-spondins are involved in the ovarian differentiation in a teleost, medaka (*Oryzias latipes*). BMC Dev. Biol. 12, 36 (2012). 10.1186/1471-213X-12-36 PMC3542121 23217106

[b21] Chakraborty T. *et al.* Differential expression of three estrogen receptor subtype mRNAs in gonads and liver from embryos to adults of the medaka, Oryzias latipes. Mol. Cell. Endocrinol. 333(1), 47–54 (2011). 21146584 10.1016/j.mce.2010.12.002

[b22] Wang D. S. *et al.* Foxl2 up-regulates aromatase gene transcription in a female-specific manner by binding to the promoter as well as interacting with Ad4 binding protein/steroidogenic factor 1. Mol. Endocrinol. 21(3), 712–25 (2007). 17192407 10.1210/me.2006-0248

[b23] Nakamoto M. *et al.* Gonadal sex differentiation and expression of *Sox9a2*, *Dmrt1*, and *Foxl2* in *Oryzias luzonensis*. Genesis 47(5), 289–299 (2009). 19298014 10.1002/dvg.20498

[b24] Gaj T., Gersbach C. A. & Barbas C. F. III ZFN, TALEN and CRISPR/Cas-based methods for genome engineering. Trends Biotechnol. 31(7), 397–405 (2013). 23664777 10.1016/j.tibtech.2013.04.004 PMC3694601

[b25] Weiss B., Davidkova G. & Zhou L. W. Antisense RNA gene therapy for studying and modulating biological processes. Cell Mol. Life Sci. 55(3), 334–58 (1999). 10228554 10.1007/s000180050296 PMC11146801

[b26] Gutschner T. Baas M. & Diederichs S. Noncoding RNA gene silencing through genomic integration of RNA destabilizing elements using zinc finger nucleases. Genome Res. 21(11), 1944–54 (2011). 21844124 10.1101/gr.122358.111 PMC3205578

[b27] Weinberg M. S. *et al.* The antisense strand of small interfering RNAs directs histone methylation and transcriptional gene silencing in human cells. RNA 12(2), 256–62 (2006). 16373483 10.1261/rna.2235106 PMC1370905

[b28] Winter J., Jung S., Keller S., Gregory R. & Diederichs S. Many roads to maturity: microRNA biogenesis pathways and their regulation. Nat. Cell Biol. 11(3), 228–34 (2009). 19255566 10.1038/ncb0309-228

[b29] Tanaka M., Kinoshita M., Kobayashi D. & Nagahama Y. Establishment of medaka (*Oryzias latipes*) transgenic lines with the expression of green fluorescent protein fluorescence exclusively in germ cells: a useful model to monitor germ cells in a live vertebrate. Proc. Natl. Acad. Sci. USA 98(5), 2544–9 (2001). 11226275 10.1073/pnas.041315498 PMC30174

[b30] Hostetler H. A. Peck S. L. & Muir W. M. High efficiency production of germ line transgenic Japanese medaka (*Oryzias latipes*) by electroporation with direct current-shifted radio frequency pulses. Transgenic Res. 12(4), 413–24 (2003). 12885163 10.1023/a:1024248300592

[b31] Kluver N. *et al.* Regulatory back-up circuit of medaka Wt1 co-orthologs ensures PGC maintenance. Dev. Biol. 325(1), 179–88 (2009). 18992736 10.1016/j.ydbio.2008.10.009

[b32] Hossain A. & Saunders G. F. The human sex-determining gene SRY is a direct target of WT1. J. Biol. Chem. 276(20), 16817–23 (2001). 11278460 10.1074/jbc.M009056200

[b33] Herpin A. *et al.* Divergent expression regulation of gonad development genes in medaka shows incomplete conservation of the downstream regulatory network of vertebrate sex determination. Mol. Biol. Evol. 30(10), 2328–2346 (2013). 23883523 10.1093/molbev/mst130 PMC3888023

[b34] Zhou L. Y. *et al.* Rspo1-activated signalling molecules are sufficient to induce ovarian differentiation in XY medaka (*Oryzias latipes*). Sci. Rep. (Accepted; *SREP-15-07077B*). 10.1038/srep19543 PMC4726049 26782368

[b35] Shah R. G., Ghodgaonkar M. M., Affar E. L. B. & Shah G. M. DNA vector-based RNAi approach for stable depletion of poly (ADP-ribose) polymerase-1. Biochem. Biophys. Res. Commun. 331(1), 167–74 (2005). 15845374 10.1016/j.bbrc.2005.03.135

[b36] Chen Y. *et al.* Nanoparticles modified with tumor targeting scFv deliver siRNA and miRNA for cancer therapy. Mol. Ther. 18(9), 1650–6 (2010). 20606648 10.1038/mt.2010.136 PMC2956922

[b37] Vilenchik M. *et al.* Antisense RNA down-regulation of bcl-xL expression in prostate cancer cells leads to diminished rates of cellular proliferation and resistance to cytotoxic chemotherapeutic agents. Cancer Res. 62(7), 2175–83 (2002). 11929841

[b38] Clayton J. RNA interference: the silent treatment. Nature 431(7008), 599–605 (2004). 15457267 10.1038/431599a

[b39] Krisnan P. *et al.* DNA constructs expressing long-hairpin RNA (lhRNA) protect *Penaeus monodon* against White Spot Syndrome Virus. Vaccine 27(29), 3849–55 (2009). 19490985 10.1016/j.vaccine.2009.04.011

[b40] Herpin A. *et al.* Inhibition of primordial germ cell proliferation by the medaka male determining gene Dmrt1bY. BMC Dev. Biol. 7, 99 (2007). 17760954 10.1186/1471-213X-7-99 PMC2034567

[b41] Nakamura S. *et al.* Hyperproliferation of mitotically active germ cells due to defective anti-Müllerian hormone signaling mediates sex reversal in medaka. Development 139(13), 2283–7 (2012). 22627284 10.1242/dev.076307

[b42] Svingen T. & Koopman P. Building the mammalian testis: origins, differentiation and assembly of the component cell populations. Genes Dev. 27(22), 2409–26 (2013). 24240231 10.1101/gad.228080.113 PMC3841730

[b43] Wu N., Yu A. B., Zhu H. B. & Lin X. K. Effective Silencing of Sry Gene with RNA Interference in Developing Mouse Embryos Resulted in Feminization of XY Gonad. J. Biomed. Biotechnol. 2012(343891), 1–11 (2012). 22500086 10.1155/2012/343891 PMC3303865

[b44] Masuyama H. *et al.* *Dmrt1* mutation causes a male to female sex reversal after the sex determination by *Dmy* in the medaka. Chromosome Res. 20(1), 163–76 (2012). 22187367 10.1007/s10577-011-9264-x

[b45] Baron D. *et al.* An evolutionary and functional analysis of Foxl2 in rainbow trout gonad differentiation. J. Mol. Endocrinol. 33(3), 705–15 (2004). 15591029 10.1677/jme.1.01566

[b46] Cutting A., Chu J. & Smith C. A. Just how conserved is vertebrate sex determination? Dev Dyn. 242(4), 380–7 (2013). 23390004 10.1002/dvdy.23944

[b47] Smith C. A. *et al.* Cloning and expression of *R-Spondin1* in different vertebrates suggests a conserved role in ovarian development. BMC Dev. Biol. 8, 72 (2008). 18651984 10.1186/1471-213X-8-72 PMC2519078

[b48] Boulanger. L. *et al.* Foxl2 is a female sex-determining gene in the goat. Curr. Biol. 24(4), 404–8 (2014). 24485832 10.1016/j.cub.2013.12.039

[b49] Caricasole A. *et al.* RNA binding by the Wilms tumor suppressor zinc finger proteins. Proc. Natl. Acad. Sci. USA. 93(15), 7562–6 (1996). 8755514 10.1073/pnas.93.15.7562 PMC38785

[b50] Bradford S. T. *et al.* A cell autonomous role for WT1 in regulating *Sry in vivo*. Human Mol. Gen. 18(18), 3429–38 (2009). 10.1093/hmg/ddp283 19549635

