# Supplementary material for: Dmy initiates masculinity by altering Gsdf/Sox9a2/Rspo1 expression in medaka (Oryzias latipes)
Source: Sci Rep. 2016 Jan 25;6:19480. doi: 10.1038/srep19480 (PMC4726206; doi:10.1038/srep19480)

Title: *Dmy* initiates masculinity by altering *Gsdf/Sox9a2/Rspo1* expression in medaka (*Oryzias latipes*)

Tapas Chakraborty [a, b, c, e, g], **Lin Yan Zhou**[a, b, d] , **Aparna Chaudhari**[e], **Taisen Iguchi**[f], **Y. Nagahama** [a, b, g *]

[a] *Laboratory of Reproductive Biology, National Institute for Basic Biology, Okazaki 444-8585, Japan*
[b] *SORST, Japan Science Technology Corporation, Kawaguchi, Saitama 332-0012, Japan*
[c] *South Ehime Fisheries Research Center, Institution for Collaborative Relations, Ehime University, Nishiura , 798-4206, Japan*
[d] *Key Laboratory of Aquatic Science of Chongqing, School of Life Science, Southwest University, Chongqing, 400715, China*
[e] *Central Institute of Fisheries Education, Mumbai, 400061, India*
[f] *Laboratory of Molecular Environmental Endocrinology, Okazaki Institute for Integrative Bioscience, National Institute for Basic Biology, Okazaki 444-8787, Japan*
[g] *South Ehime Fisheries Research Center, Institution for Collaborative Relations, Ehime University, Matsuyama 790-8577, Japan*

Short Title: Molecular mechanism of *Dmy* associated sexuality in medaka

* Corresponding author at: South Ehime Fisheries Research Center, Institution for Collaborative Relations, Ehime University, Matsuyama 790-8577, Japan.

Phone: (+ 81) 895 73 7112. *E-mail address:* nagahama.yoshitaka.mh@ehime-u.ac.jp

Key words: Dmy; sex reversal; loss-of-function; F3 progeny; GSDF; Sox9; Masculinity; Medaka

Supporting Information:

1. **Supplementary materials and methods**

2. **Supplementary Table 1: Summary of GFP knockdown using *olvas-eGFP* transgenic medaka at F_0 generation.**

3. **Supplementary Table 2: Summary of shDNA dependent *Dmy* knockdown in QurtE medaka.**

4. **Supplementary Table 3: Summary of *Dmy* knockdown in medaka.**

5. **Supplementary table 4: Generation and evaluation of Dmy-AS founder transgenics**

6. **Supplementary Table 5: Egg production characteristics of *Dmy* knockdown fish.**

7. **Supplementary Table 6: List of major primers used in this study.**

8. **Supplementary Figure 1: Construction of *Dmy* knockdown plasmid and *in silico* analysis of efficacy.**

9. **Supplementary Figure 2: Possible mechanism of vector based AS DNA dependent AS RNA machinery *in vivo*.**

10. **Supplementary Figure 3: Effect of *Dmy* knockdown on gonadal gene profile in adults.**

11. **Supplementary figure 4. *In vivo* evaluation of knockdown efficiency of F_2 transgenics.**

12. **Supplementary figure 5. *In vivo* chromatin immunoprecipitation analysis of candidate *Dmy* targets.**

13. **Supplementary Figure 6: Effect of *Gsdf* and *Sox9a2* overexpression on germ cell proliferation and reversal of *Dmy*-KD phenotype.**

14. **Supplementary figure 7. Schematic diagram showing the breeding and mating strategies of Dmy-KD transgenic line.**

Supplementary Materials and Methods

Plasmid constructs. The AS (Antisense) RNA expression constructs were designed and evaluated using the online tool E-RNAi. Several constructs of different length (200-320bp) were selected, amplified, evaluated *in vitro* before starting the actual experiment. A selected primer pair carrying *Xba*I and *EcoR*V sites (Supplementary table 5) was used to amplify a 220-bp region of the *Dmy* gene (Supplementary Fig. 1) from a plasmid containing the complete *Dmy* ORF (a gift from Dr. M. Matsuda, Japan). A 359-bp sequence of the *Gfp* gene was also amplified from the plasmid pCMV-GFP (Dr. Krishnan, India) using specific primers with the same RE sites (Supplementary table 5). The amplicons were cloned under the control of the CMV early promoter in pcDNA3.1 (+) (Invitrogen, USA) in the AS orientation. The constructs were transformed into *E. coli* (XL1 blue) and the positive clones were confirmed by sequencing. The sequences were also analysed for secondary structures *in silico* using RNA*fold* online software (http://rna.tbi.univie.ac.at/cgi-bin/RNAfold.). The recombinant plasmids were designated as pmDMY-AS and pEGFP-AS. Plasmid DNA used for downstream experiments was purified using a plasmid purification kit (Qiagen, Germany). A schematic representation of the AS RNA expressing construct is given in supplementary Fig 1.

The complete *Dmy* ORF (820 bp) was cloned into the pCDNA 3.1 histopo T-vector using ORF-specific primers (Supplementary table 5). A clone that contained the insert in the right orientation was selected after sequencing. The recombinant plasmid was named pmCMV-DMY. Similarly, complete ORFs of *Wt1a/b*, *Ad4bp/Sf1, Sox9a2* and *Gsdf* were also amplified (from previous constructs in the pGEMTeasy vector), cloned into pCDNA 3.1 histopo T-vector, sequenced to confirm the correct orientation and named as pCMV-Wt1a/b, pCMV-Sf1, pCMV-Sox9a2 and pCMV-Gsdf, respectively. A 300 bp *Dmy* promoter fragment was isolated using specific primer set and cloned in pGL3 basic vector (Promega, USA) using Infusion-cloning kit (Clonetech, USA). Stop codon-less *Gsdf* and *Sox9a2* ORFs were inframed with Cherry (from pmCherryN1 vector) and cyan (from

pamCyanN1 vector) ORF, respectively, and cloned into a pCS2 vector (a gift from Dr. Ogino, NIBB, Japan) using Infusion cloning kit (Clonetech, USA). All the genes used for real-time PCR and *in situ* analysis were cloned into pGEM T Easy vector.

Gene knockdown in Cos7 cells. Knockdown of *Dmy* expression by the pmDMY-AS construct was tested in Cos-7 cells with no endogenous expression of *Dmy*, *Dmrt1, Sox9a2, Ad4bp/Sf1* or *Gsdf.* Cos-7 cells were seeded in 24-well plates, at 5 X 10^5 cells/well, containing Dulbecco's modified Eagle's Medium (Sigma, USA) supplemented with 10% charcoal/ dextran treated foetal bovine serum (Hyclone, USA). The cells were incubated for 24 h at 37 ^{0}C with 5% CO_2 and co-transfected with 100 ng of pmCMV-DMY and 5/ 10/ 100 ng of the pmDMY-AS construct using Fugene-6 transfection reagent (Roche Diagnostics, Switzerland) following the manufacturer's instructions. Cells transfected with only pmCMV-DMY served as positive controls while the negative controls were treated with only the transfection reagent. The cells were grown for 96 h after which total RNA was isolated and target transcripts quantified by real-time PCR as detailed later. The transfections were performed in triplicate at one time point and then repeated twice later. In separate experiments, cells were also transfected with pCMV-DMRT1, pCMV-SOX9a2, pCMV-SF1 and pCMV-GSDF, and the effect of pmDMY-AS on their expression was also quantified by real-time PCR. The transcript copy numbers are shown as the mean ± SEM and the dose-response data was computed using Statview v. 5 (SAS institute Inc, Cary NC).

Experimental animals. The QurtE strain of medaka was mostly used for this study. This strain expresses a male-specific leucophore that allows easy sexing of fish. Another strain called the *olvas-eGFP* transgenic medaka was also used. This strain carries the *Gfp* gene under *vasa* promoter and is reported to express GFP fluorescence in germ cells in both males and females[1]. All the fish were maintained at 26 ± 2 ^{0}C under a 14 h light and

10 h dark cycle. Eggs were collected within 30 min of fertilization and incubated in distilled water (milli-Q) containing antifungal solution (Methylene blue, 0.0001%) at 26 $\pm$ 2^{0}C. Brooders and juveniles were fed with fresh artemia, while larvae were given artificial feed. Classification of developmental stages was done according to the descriptions of Iwamatsu[2]. All *in vivo* experiments were conducted following protocols and procedures approved by Institutional Animal Care and Use Committee at the National Institute for Basic Biology, Japan.

Knockdown of *Dmy* expression in medaka embryos. One-two cell embryos of the QurtE strain of medaka were electroporated with the pmDMY-AS plasmid construct (1 μg/ml) in 1X HBS buffer, pH 7.5[3]. Electroporation was carried out in a Cuy21 edit type electroporator (Bex, Tokyo) at 24V, with 9 millisecond (ms) pulse, 900 ms pause for 3 times. Embryos electroporated with-HEPES buffered saline (HBS) alone and those electroporated with pCMV-GFP constructs served as controls. peGFP-AS plasmids were electroporated into one-two cell embryos of *olvas-eGFP* transgenic medaka to evaluate the knockdown of GFP expression.

Sampling. Electroporated male embryos of the QurtE strain of medaka were randomly sampled at 5 days after fertilization (daf) and 0, 5, 10, 20, 50 and 90 days after hatching (dah) in order to check the gonadal morphological changes and further confirming the gene knockdown effects at both genetic and phenotypic levels. Minimum of 10 fish were sampled at each time point. The genetic sexes of all fish were confirmed by *Dmy*-genomic PCR[4]. Electroporated *olvas-eGFP* transgenic medaka were periodically observed for any fluorescence reduction and morphological defect until hatching, and all the fish of 10 dah were culled and fixed for histological examination of the gonads. All fish were also sexed accordingly[4]. 10 adults of control XY and DmyKD-XY (Dmy-AS (+)) groups were dissected and different tissues were separately collected to prepare the

tissue distribution samples. The early stage samples (from Stage 20 to 39) were initially separated based on leucophore expression and later the individuals were separately screened using *Dmy* genomic PCR. The *Dmy* positive and negative samples (10 individual each) of the same stage were separately pooled together. Other earlier stage RNA samples (from Stage 4 to 19) were collected from monogametic populations of XX and XY. Three different pools of samples were prepared for reproducible results.

Histology and *in situ* hybridisation. Whole fish samples at 0, 5, 10 and 50 dah and gonads of adults were fixed in Bouin' fixative, embedded in paraffin, sectioned at 5 μm and subjected to standard haematoxylin-eosin (HE) staining. Paraformaldehyde (4%) fixed, paraffin embedded samples (10 dah and adult) were used for *in situ* hybridisation. Hybridisation was done using sense and anti-sense digoxigenin-labelled RNA probes transcribed *in vitro* with a RNA labelling kit (Roche, Germany) from their respective plasmids. Briefly, the sections were deparaffinised, hydrated and treated with proteinase K (10 μg/ml, Roche) and then hybridised with sense or anti-sense DIG-labelled RNA probes at 58 ^{0}C for 22 h. Hybridisation signals were then detected using alkaline phosphatase-conjugated anti-DIG antibody (Roche) and NBT as described previously[5]. Whole mount *in situ* hybridisation was carried out using previously published protocols[6].

Quantification of changes in gene expression by real-time PCR. Changes in gene expression were quantified using the ABI Prism 7000 sequence detection system (Applied Biosystems, USA). Total RNA was isolated from cells, embryos, and gonads, using RNeasy Mini kit (Qiagen, Germany). cDNA synthesis was carried out using Quantitect RT PCR kit (Qiagen) from 100 ng of total RNA. The first strand cDNAs were diluted to 100 μl for subsequent use. Gene-specific RT-PCR was performed using SYBR green master mix (Applied Biosystem, USA) and 5 ng of cDNA, according to the manufacturer's instructions. The real-time PCR primers are listed in Supplementary table

5. The PCR conditions included an initial denaturation at 94 ^{0}C (2 min) followed by 40 cycles at 94 ^{0}C (30 s) and 60 ^{0}C (1 min). *Ef1α* or *Rps18* was used as the internal control. The absolute transcript copy number of each gene was determined with the help of appropriate standard curves and normalized with the *Ef1α/ Rps18* copy numbers in each sample. The reported values are average of sample triplicates.

Assessment of the trans-generational knockdown effect. The QurtE embryos injected with pmDMY-AS were grown to adulthood. Based on secondary sexual characters and the presence of leucophores, genetic males that sex reversed to females were identified and mated with normal males to assess their breeding behaviour and mating performance. Successfully mated XY females were used for the production of F_1 progeny (Supplementary figure 7). Caudal fin clips from XY females of the F_0 and F_1 generations were used for genomic DNA isolation[4]. The genome integration of the pmDMY-AS construct was tested using a primer pair that amplified a portion of the vector and *Dmy* AS sequence (Supplementary table 5) and then confirmed by sequencing. Aliquots of the same genomic DNA samples were analysed by real-time PCR to estimate the number of integrated copies. The breeding experiment was similarly performed with F_1 and F_2 fish, and the offsprings were histologically examined at 10 and 20 dah.

Characterisation of small RNAs. To determine the AS plasmid associated mechanism of RNA suppression *in vivo*, total RNA (enriched with small RNA) was isolated from the adult gonads of F_1 progeny, using the miRNeasy mini kit (Qiagen), following the manufacturer's protocol. Small RNAs were separated using 15% denaturing polyacrylamide gel electrophoresis. A Northern blot analysis was performed using a digoxigenin-labelled AS *Dmy* probe following the standard protocol. In a separate experiment, RNA bands ranging from 18-28 nucleotides as well as 60-70 nucleotides were excised from gels, and purified using RNA extraction buffer of the DynaExpress

miRNA cloning kit (Bio Dynamics, Japan). 5` and 3` linkers were added to the purified small RNA and cDNA was produced, according to the manufacturer's instructions. The cDNAs were PCR amplified and cloned into the pGEMTeasy vector (Promega, Japan). Both colony PCR and dot blots (using dig-labelled AS *Dmy* probes) were performed to screen the inserts and the positive clones were sequenced.

Promoter analysis. The promoter analysis was performed using a previously described protocol[7]. Briefly, HEK293 cells were seeded in 24-well plates, at 5 X 10^5 cells/well, containing Dulbecco's modified Eagle's Medium (Sigma, USA). After 24 hours, the cells were transfected with pGL3-DMY-luciferase plasmid and either one of pcDNA3.1-WT1a/b (both +/-KTS) plasmids, at different concentration (0-200 ng/well) in triplicates. The luciferase assay was performed after 48 hours of transfection. The experiment was repeated thrice for reproducibility. The Ol32 cells were similarly prepared in L-15 medium (Sigma, USA) and co-transfected with pGL3-Gsdf or pGL3-Sox9a2 and pcDNA3.1-Dmy to assess the *Dmy* associated *Gsdf* and *Sox9a2* promoter activity.

***In vivo* Chromatin Immunoprecipitation analysis of *Dmy*.** Binding sites for *Dmy* were identified using the matrix as provided[8], together with the Regulatory Sequence Analysis Tools portal: RSat (http://rsat.ulb.ac.be/rsat/, last accessed February 15, 2015), weight score $\geq$ 1, p-value $\leq$1. For *in vivo* chromatin immunoprecipitation (ChIP), the ChIP expression-shearing kit (Active motif) was used according to the manufacturer's instructions, using 20 mg of embryonic tissue samples from *Dmy-Gfp* mRNA injected fish (50 embryos for each) and GFP monoclonal antibody (3 mg, AbCAM). The *Dmy-Gfp* plasmid was constructed by sequentially fusing *Dmy*-5`UTR, *Dmy*-ORF (stop codon less), *eGFP*, and *Dmy*- 3`UTR into a pCS2 vector using infusing cloning kit (Clonetech) and RNA was synthesized with mMESSAGE mMECHINE SP6 kit (Ambion, USA)

following polyA addition with Poly-A tailing kit (Ambion) After tissue disaggregation and cell re-suspension, DNA was sheared according to the manufacturer's protocols. The ChIP procedure using *Dmy-Gfp* mRNA embryos was validated as described earlier[8].

Rescue of *Dmy* knockdown effects. pmDMY-KD XY adult females (F_3 generation) were mated with *olvas-eGFP* XY males. The resulted progenies were screened for integration, grown to maturity and backcrossed with *olvas-eGFP* XY fish for 3 consecutive generations and *olvas-eGFP-Dmy*-KD transgenic line was created. The F_3 generation fish developed from this line were used for rescue analysis. Briefly, the *Gsdf*-mcherry and *Sox9a2*-cyan mRNA, flanked by 5' and 3' untranslated regions of respective genes, were synthesized with mMESSAGE mMECHINE SP6 kit (Ambion, USA) following polyA addition with Poly-A tailing kit (Ambion). Control RNAs (only mcherry and cyan, flanked by both 5' and 3' UTRs of *Gsdf* and *Sox9a2,* respectively) were similarly prepared. The purified RNA was injected in one-two cell stage embryos of *Dmy*-KD fish @ 1ng/ul. Both reporter gene expression (mcherry and cyan, whatever applicable) and gonad development were monitored using Axioplan2 imagining (Zeiss, USA) confocal microscope for each embryo separately. The embryos showing reporter gene expression were then grown separately until 10 dah in a 24 well dish @ 1 embryo/well and either fixed/ preserved for histological/ real-time PCR analysis or grown further to assess morphological sex change. Few of the manipulated fish caudal fin bud clips were used for genomic integration and genomic sex determination. Only the XY embryos having *Dmy*-KD insert (confirmed by integration PCR) were included in the analysis.

Data analysis. Statistical differences in relative mRNA expression between experimental groups were assessed by One Way ANOVA, followed by Tukey's test. All statistical analyses were performed using SPSS, version 18 software. All experimental

data are shown as the mean ± SEM. Differences were considered statistically significant at $p<0.05$, if not otherwise mentioned. Specially used data analysis methods are described in the respective sections.

References

1. Tanaka, M. Kinoshita, M. Kobayashi, D. & Nagahama, Y. Establishment of medaka (*Oryzias latipes*) transgenic lines with the expression of green fluorescent protein fluorescence exclusively in germ cells: a useful model to monitor germ cells in a live vertebrate. *Proc. Natl. Acad. Sci. USA*, **98**(5), 2544-9 (2001).
2. Iwamatsu, T. Stages of normal development in the medaka *Oryzias latipes*. *Mech. Dev*, **121**(7-8), 605-18 (2004).
3. Hostetler, H. A. Peck, S. L. Muir, W. M. High efficiency production of germ line transgenic Japanese medaka (*Oryzias latipes*) by electroporation with direct current-shifted radio frequency pulses. *Transgenic Res*, **12**(4), 413-24 (2003).
4. Matsuda, M. *et al*. DMY is a Y-specific DM-domain gene required for male development in the medaka fish. *Nature*, **417**(6888), 559-63 (2002).
5. Chakraborty, T. *et al*. Estrogen receptors in medaka (*Oryzias latipes*) and estrogenic environmental contaminants: an *in vitro–in vivo* correlation. *J. Steroid Biochem. Mol. Biol*, **123**(3-5), 115-21 (2011).
6. Inohaya, K. *et al*. Temporal and spatial patterns of gene expression for the hatching enzyme in teleost embryo *Oryzias latipes*. *Dev. Biol,* **171**, 374-85 (1995).
7. Wang, D. S. *et al*. Foxl2 up-regulates aromatase gene transcription in a female-specific manner by binding to the promoter as well as interacting with Ad4 binding protein/steroidogenic factor 1. *Mol. Endocrinol*, **21**(3), 712-25 (2007).
8. Herpin, A. *et al*. Divergent expression regulation of gonad development genes in medaka shows incomplete conservation of the downstream regulatory network of vertebrate sex determination. *Mol. Biol. Evol.* **30**(10), 2328–2346 (2013).

Supplementary tables:

Supplementary table 1: Summary of GFP knockdown using *olvas-eGFP* transgenic medaka at F_0 generation.

Stage and type of sampling	Analysis method	Number of fish sampled	Number of fish changed
0 dah	Microscopic GFP fluorescence observation[1]	10	6
10 dah	Real-time PCR[2]	10	10
	Microscopic GFP fluorescence observation[1]	10	7
Adult (4 months)	Secondary sexual character [3]	20	0

Note: Numbers in superscript indicate the biological end points analysed using the samples. 1. Change in *olvas-eGFP* expression pattern; 2. Change in germ cell number and occurrence of meiosis/ meiotic arrest; 3. Occurrence of fan like anal fin and forked dorsal fin in XX, and tapering anal fin and fused dorsal fin in XY fish.

Supplementary table 2: Summary of shDNA dependent *Dmy* knockdown in QurtE medaka.

Sampling group	Analysis method	Number of fish sampled	Mitotic cell number ± SE	Meiotic cell number ± SE	Number of fish changed	% change
**pmCMV-DMYsh1**	QPCR[1]	10			10	100
	HE[2]	10	101±27.6	0	2[3]	20
**pmCMV-DMYsh2**	QPCR[1]	10			9	90
	HE[2]	10	104± 14.4	0	4[3]	40
**pmCMV-DMYsh3**	QPCR[1]	10			10	100
	HE[2]	10	119 ± 11.8	1 ± 0.8	2[3], 1[4]	20
**pmCMV-DMYsh1 +sh2**	QPCR[1]	10			8	80
	HE[2]	10	210 ± 31.7	3 ± 2.9	5[3]	50
**pmCMV-DMYsh1 +sh3**	QPCR[1]	10			10	100
	HE[2]	10	230 ± 42.1	6 ± 4.2	5[3], 3[4]	50
**pmCMV-DMYsh1+sh2+sh3**	QPCR[1]	10			10	100
	HE[2]	10	390 ± 21.2	19 ± 9.2	8[3], 6[4]	80

Note: Numbers in superscript indicate the biological end points analyzed using the samples. 1. Change in gene expression; 2. Change in germ cell number and occurrence of meiosis/ meiotic arrest; 3. Mitotic germ cell population; 4. Meiotic germ cell population.

Supplementary table 3: Summary of *Dmy* knockdown in medaka.

Stage of Sampling	Analysis method	Number of sample (N)	Number of fish with abnormal characters
**F_0 generation**			
**0 dah**	Real-time PCR[1]	10	8
	Histology[2]	30	7
**10 dah**	Real-time PCR[1]	10	10
	Histology[2]	10	9
	ISH[3]	10	6
**50 dah**	Histology[2]	10	2*, 5[#]
**Adult**	Secondary sexual character[4]	10	7
	Breeding behaviour[5]	5	5
	Gene integration[6]	5	2
**F_1 generation[$]**			
**10 dah**	Histology[2]	361	60
	ISH[3]	10	3
**Adult**	Secondary Sexual Characters[4]	50	15
	Genome integration[6]	12	3
	Breeding behaviour[5]	3	3
**F_2 generation**			
**10 dah**	Histology[2]	10	3
	ISH[3]	10	4
**Adult**	Secondary sexual character[4]	30	12
	Breeding behaviour[5]	12	4
**F_3 generation**			
**2 daf**	Histology[2, 7]	10	4
**0 dah**	Rescue with *Sox9a2*[2,8]	20	0
	Rescue with *Gsdf*[2,8]	20	0
	Rescue with *Sox9a2* and *Gsdf* coexpression[,2,8]	10	10
**Adult**	Secondary sexual character[4]	42	41
	Breeding behaviour[5]	5	5

Note: Numbers in superscript indicate the biological end points analysed using the samples. 1. Change in gene expression; 2. Change in gonadal structure, germ cell number and occurrence of meiosis/ meiotic arrest; 3. Changes in cellular localisation of different gene; 4. Occurrence of tapering anal fin and fused dorsal fin; 5. Mating Characteristics i.e. chasing, dancing, coiling, and pressing; 6. Positive PCR amplification of AS probe using a vector and gene specific primer pair; 7. Differences in *Wt1* expression; 8. Gonadal structure at 50dah; '*' Partial sex change and occurrence of testis ova; '#' Complete sex change

and occurrence of complete ovary; '[$]' combined profiles of two founder. The breeding behaviour and gene integration checks were performed with XY female fish (depending on secondary sexual characters).

Supplementary table 4: Generation and evaluation of Dmy-AS founder transgenics

Crossing method:	Control XY ♂ **X** Dmy-KD-XY♀	
Characters	Founder 1	Founder 2
Number of eggs laid in first three week from 5 month of age	211	187
Hatched embryos after 7 days of fertilization	189	172
Dmy-AS(-) XX ♀ embryo at 10 dah*	53	45
Dmy-AS(+) XX ♀ embryo at 10 dah*	47	48
Dmy-AS(-) XX ♂ embryo at 10dah*	0	1
Dmy-AS(+) XX ♂ embryo at 10dah*	0	0
Dmy-AS(-) XY ♀ embryo at 10 dah*	**0**	**0**
Dmy-AS(+) XY ♀ embryo at 10 dah*	**32**	**28**
Dmy-AS(-) XY ♂ embryo at 10 dah*	38	36
Dmy-AS(+) XY ♂ embryo at 10 dah*	19	14

The sex reversals are indicated in bold. '*' Genotyping, integration specific PCR and histology of individual embryo was carried out to determine the gonadal sex.

Supplementary table 5: Egg production characteristics of *Dmy* knockdown fish.

Groups	Age (month)	Progeny status			
		**F_2**	**F_3**	**F_4**	**F_5**
**Control**					
	4	125.7±5.0	134.0±5.3	121.3±5.4	139.0±4.2
	5	289.0±4.7	274.0±6.2	274.0±5.9	260.0±4.2
	6	331.0±4.0	347.3±4.7	340.7±4.3	355.0±6.7
	7	374.0±14.8	369.7±2.2	336.0±5.9	362.3±3.9
	8	359.7±8.5	334.0±4.4	389.0±4.5	328.7±3.3
	**Total**	**1479.3±22.2**	**1459.0±20.0**	**1461.0±22.5**	**1445.0±19.4**
**DMY- KD**	4	119.7±4.8	115.7±4.8	125.7±4.5	119.7±1.9
	5	235.0±4.5	254.3±6.0	223.3±6.6	230.7±6.6
	6	321.0±4.0	364.7±4.5	364.0±5.5	341.0±3.5
	7	365.0±13.5	359.0±5.7	378.0±19.8	385.7±2.9
	8	386.3±6.0	341.3±5.0	318.0±5.3	354.0±10.1
	**Total**	**1427.0±6.0**	**1435.0±14.6**	**1409.0±16.3**	**1431.0±8.7**
**Significanc (p<0.05)**	**NS**	**NS**	**NS**	**NS**	**NS**

Supplementary table 6: List of major primers used in this study.

Primer name	Sequence	Purpose
Dmy 24 F	TCCTATTATGGAAACCTGCACAACTAC	*Dmy* real-time PCR
DMY 26R	GAAGGAGTGCATGCGGTACTG	*Dmy* and *Dmrt1* real-time PCR
Dmrt1 25 F	TCCTCCTACTATGGAAACCTGTACCA	*Dmrt1* real-time PCR
Gsdf realtime F	GGGCTGGACACTATTCGAGA	
Gsdf realtime R	CATGACACAGAGGAGCTGGA	
Sf1 realtime F	AGCTGCTACTCTGGAAACGA	
Sf1 realtime R	ACTGGCAATCTTCTTGGCAGC	
GFP realtime F	CGACAACCACTACCTGAGCA	
GFP realtime R	GAACTCCAGCAGGACCATGT	
Olvas realtime F	CCCAAAGTGACCTACATC	
Olvas realtime R	AAGTTGATGCCCATCTTG	Measuring Gene expression by real-time PCR
Foxl2 realtime F	AAACCTGCTACTCTGGACGC	
Foxl2 realtime R	AGTCAAATCTTCTTGATTC	
Fig1a realtime F	TGTACTGCTGCATCGAGAAGTA	
Fig1a realtime R	ATGCTGCAACACCAGTCTAGT	
Spo11 realtime F	TCGATTCTGGTGCCGTCTTCT	
Spo11 realtime R	ATGCTGAAGGTTTCTCGCAGG	
Rspo1 realtime F	TGCAACACCAGTCTAATG	
Rspo1 realtime R	TTCTGGTGCCGTCTTCTAGG	
Cyp19a1 RT F	AGCTTATTTTGCCCAAGGCC	
Cyp19a1RT R	TTGAGCAGCAGGAGCATGAAA	
Gsdf ORF F	ATGTCTTTGGCACTCATT	ORF amplification, *in situ* probe preparation, synthetic RNA preparation
Gsdf ORF R	CTACTTTTTGCAGGGCTGCT	
Sf1 ORF F	AGCAAGGGTGTGAGGAG	
Sf1 ORF R	TTTTTGCAGGGCTGCT	
Dmy ORF F	CCGCGGGAGCTCATGAGCAAGGAGAAGCAGTGC	ORF amplification, *in situ* probe preparation
Dmy ORF R	GGATCCGAATTCTGGAGTTGGCCGGGAAGACG	
Spo11 F	GATGCAAGGAGAGAGTT	
Spo11 R	ATACTCAGCTGTTTGGGTCACA	
Foxl2 F	TGCACCTGACACCAGTCT	
Foxl2 R	TAGACAACACCGAGTCTG	
Olvas F	AAGAGCTCCCAGCAAGGC	
Olvas R	TCGGAGCTCATGAGCAAGG	
Rspo1 F	TGCAACACCAGTCTAATG	*In situ* probe preparation
Rspo1 R	TAGACTGCCCGTCATG	
Fig1α F	ATGAAGGTGCCAGAGGCGGAAT	
Fig1α R	TTAATCCCTCGAAGCTTGATCG	
Dmy-as-Xba1 F1	TCTAGAGGAAACCTGTACAACTACCA	
Dmy-as-Eco RV R1	GATATCCATGGGTGGAGGTGAGGCT	Amplification of *Dmy* antisense region, integration check
M13 F	GATATCCGAGCATCTCCAGTAGGAGG	
M13 R	TCTAGATCTACAGCATGAAGTGCAA	Colony PCR,

T7	GATATCGACCCTCCATACTGAAGGA	Sequencing, *in situ* probe preparation
T3	TCTAGACTCTTTGTTCTGGCAAAGCC	Sequencing, insert check
Sp6	GATATCGGTTGCAGGGCAGATGTAGT	
Bgh R	TCTAGAATGGGAACCACTTTGGACTC	
pcDNA 3.1 807 F	GATATCAGCTGAAGATGGTTGGGTTG	
PCDNA 3.1 1127 R	ATGAAGGTGCCAGAGGCGGAAT	
Dmy genomic F	TTAATCCCTCGAAGCTTGATCG	Genomic PCR for sorting of sex
Dmy genomic R	TCTAGAAGGAGGAGCTTGGGATTTGT	
EF1α F	GATATCCGTCCCTCCACAGAGAAGAG	Internal control for Real Time PCR
EF1α R	TCTAGACTCTTCTCTGTGGAGGGACG	
Dmy5utrF	TCTTTTTGCAGGATCGAAAGACTGTTCTCCGGTAAATTGACG	Construction of *Dmy* overexpressing plasmid
DmyORFR	CTCACCATTTTGGTTTCACTGCTCATGGAGTTGG	
EGFP-F	AACCAAAATGGTGAGCAAGGGCGAG	
EGFP-R	GCTTTTCCTTACTTGTACAGCTCGTCCATGCC	
Dmy3utrF	CAAGTAAGGAAAAGCTGCGAGGC	
Dmy3utrR	CGAATCGATGGGATCGACCATCTCATTTTTTATTCTTGATTTTTCACTTTCATCT	
Gsdf geno-F1	CTGCCGGATCATTCTCCTTG	Real-time analysis of ChIP DNA
Gsdf-geno-R1	GGGCCTGGGAACCTGAGT	
Sox9a2geno-F1	GCGCACGAGGTTTTATTGAGT	
Sox9a2geno-R1	TCTCTTTCCAGGCTCCTTAATG	
Rspo1 geno-F1	ATGGTGTGCAGTCTGGTTTAAT	
Rspo1 geno-R1	ATAAACTTTGCAATCATAACTGTT	

Supplementary figures:

Supplementary figure 1. Construction of *Dmy* knockdown plasmid and *in silico* analysis of efficacy. The *Dmy*-knockdown plasmid was constructed on a pcDNA3.1 (+) vector backbone (A). The AS fragment/probe, predicted to have the best secondary AS RNA structure (B), was inserted in a reverse orientation into the pcDNA 3.1(+) vector to generate the knockdown plasmid.

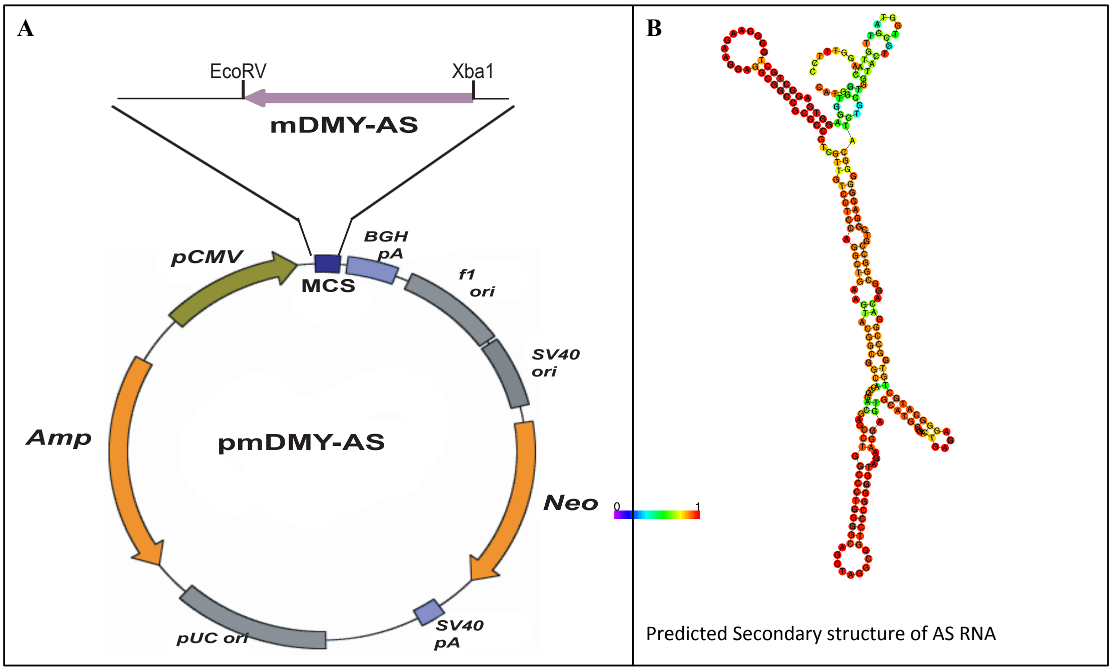

Supplementary figure 2. Possible mechanism of vector based AS DNA dependent AS RNA machinery *in vivo*. Northern blotting was used to identify the *Dmy* specific RNA degradation (A). Lane 1, 2, 3, 4, 5 in northern blot represents small RNAs from adults of *Dmy-KD* F_1 XX, *Dmy-KD* F_1 XY, wild QurtE medaka XX, wild QurtE medaka XY and negative control (only RNA loading buffer), respectively (A). The maximally expressed lower molecular weight RNAs, marked in the chemo-luminescent graph as per their sizes i.e. 60 -70 mer and 18-28 mer, were used for further isolation and small-RNA library construction. The original *Dmy* AS sequence (B_1) was used as a reference to find out the *Dmy* specific small AS RNAs in the small-RNA libraries constructed using 60-70 (total clones= 4261) and 18-28 (total clones = 6176) mer purified RNAs. Chromatograms of two different 70 mer (out of total 4261 clones) and three different 18-28 mer (out of total 6176 clones) are presented in B_2 and B_3, respectively. The pink boxes in B_2 panel represents the sequences of 18-28 mer RNAs obtained from the 18-28 mer library. (C) Schematic representation of hypothetical pathway of knockdown *in vivo*.

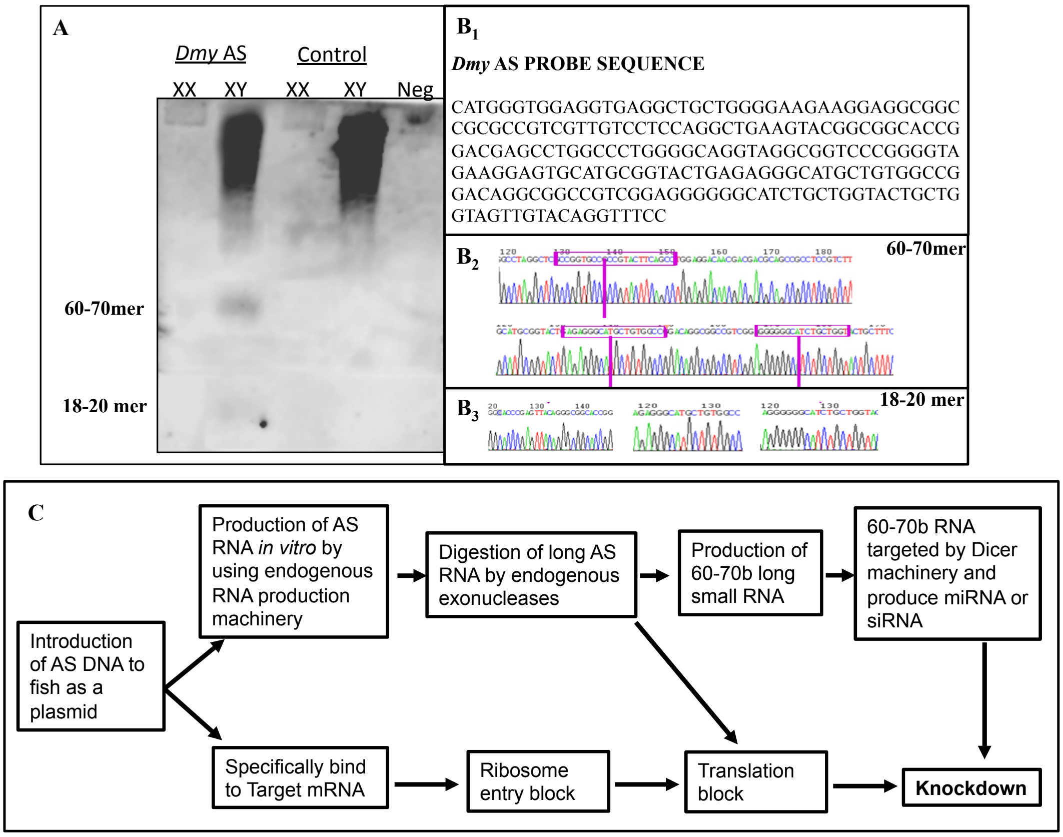

Supplementary figure 3. Effect of *Dmy* knockdown on gonadal gene profile in adults. Data are presented as both individual values (with dots and triangles) and mean of 15 XY individuals (white and grey columns for control and knockdown groups, respectively). Inset: representative figure for genotyping of the fish used for real-time PCR, where the upper and lower band represents *Dmrt1a* and *Dmy* specific amplification, respectively; M-marker.

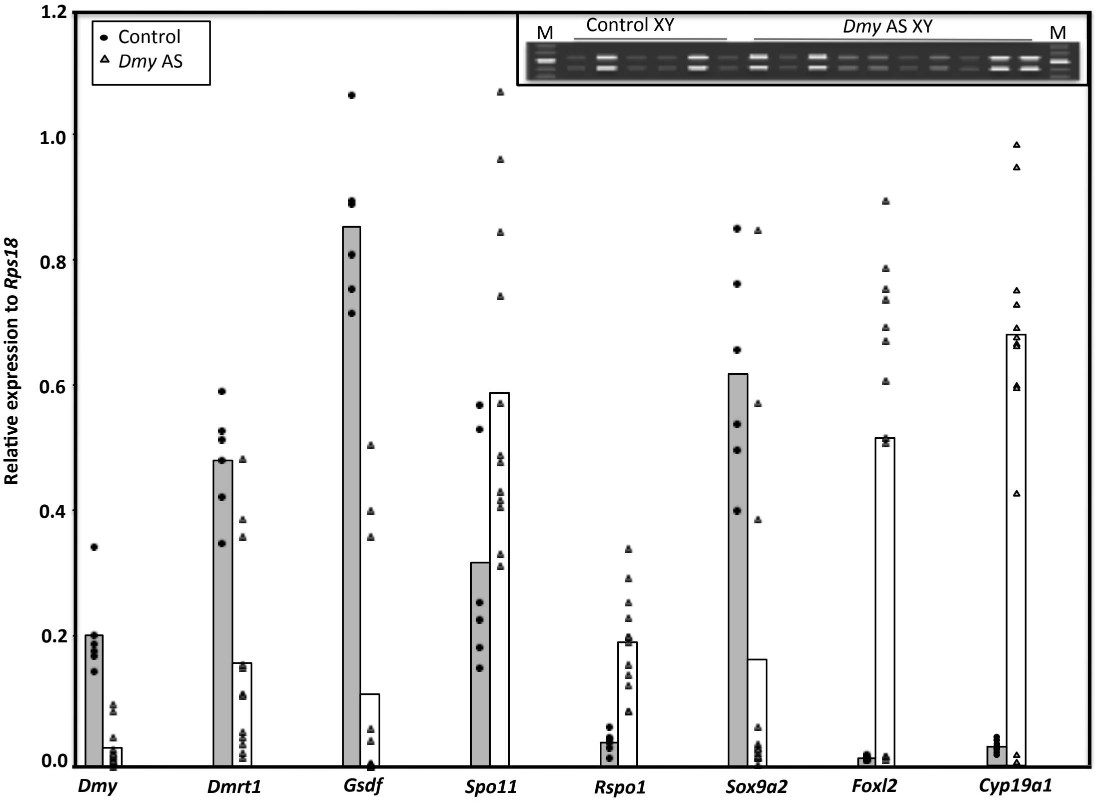

Supplementary figure 4. *In vivo* evaluation of knockdown efficiency of F2 transgenics. The distribution of antisense RNA in different tissues were assessed by AS-RNA specific primers to evaluate the germ line transmission in Dmy-KD (Dmy-AS(+)) 5 month adults (A). The same samples were further checked for *Dmy* transcription, compared with control XY and plotted in graphs as relative abundance to internal control (B). 20, 50 and 90 dah Dmy-AS(+) XY individuals were evaluated for *Dmy* (C) and *Dmrt1* (D) transcriptional silencing. Note: Each column in the graph represents mean ± SEM of 10 individuals and letters (a, b, c, etc.) above the bars indicate that these groups differ significantly ($p<0.05$) from each other.

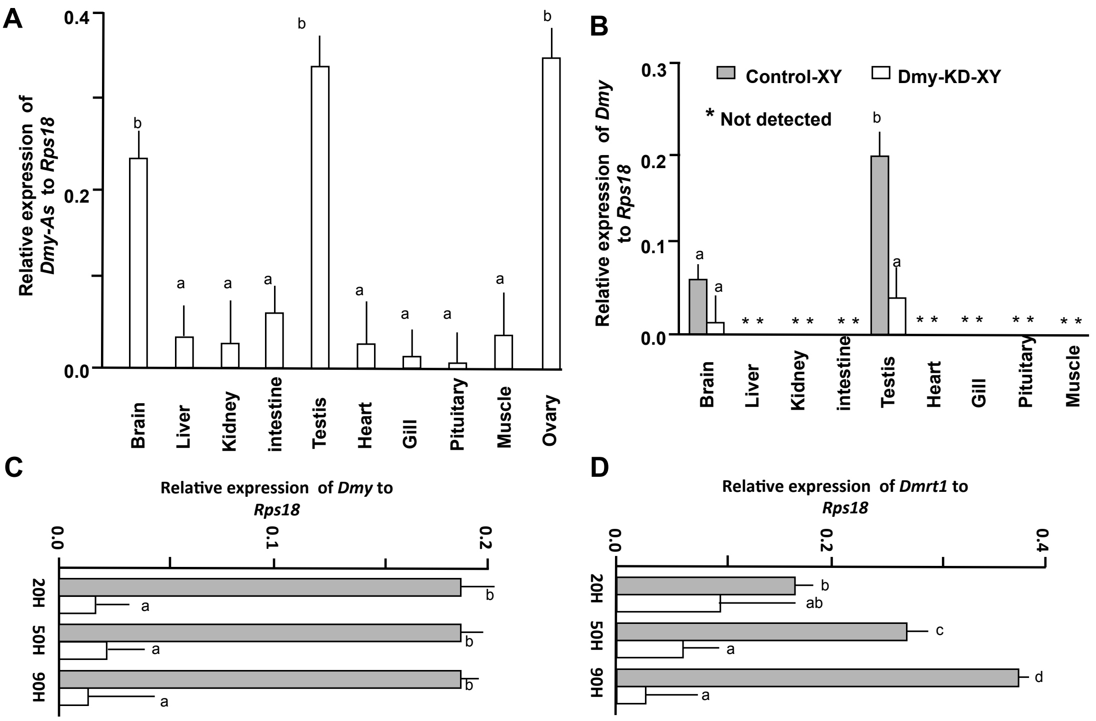

Supplementary figure 5. *In vivo* chromatin immunoprecipitation analysis of candidate *Dmy* targets. Relative expression to control input was calculated using realtime PCR and plotted on the graph. Data are presented as means ± SEM of 4 separate experiments and significances ($p<0.05$) are denoted by different letters.

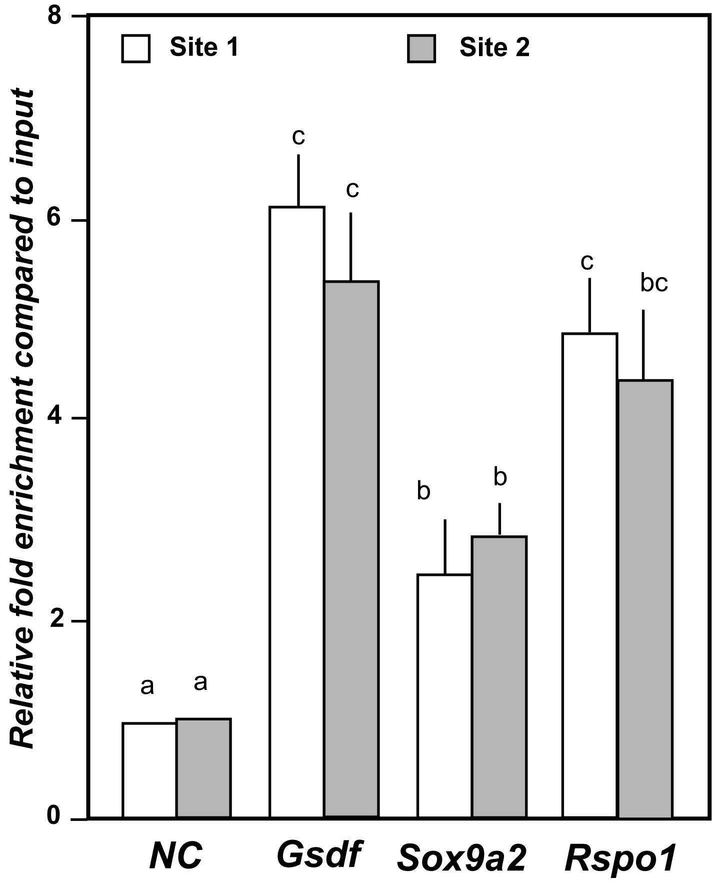

Supplementary figure 6. Effect of *Gsdf* and *Sox9a2* overexpression on germ cell proliferation and reversal of *Dmy*-KD phenotype. Germ cell (green colour) proliferation and clustering patterns of *olvas-eGFP* transgenic medaka (both control and *Dmy*-KD) gonads at S39 (1 day before hatching) were assessed using confocal microscope. Isolated (type-I) and clustered (type- II) germ cells are marked with * and #, respectively. (A-D) *Olvas-eGFP*-control-XX and (E-H) *olvas-eGFP-Dmy*-KD-XY embryos. Note: N= 9 per group and control RNAs were prepared by mcherry/cyan fusion with *Gsdf* or *Sox9a2* 3` UTR.

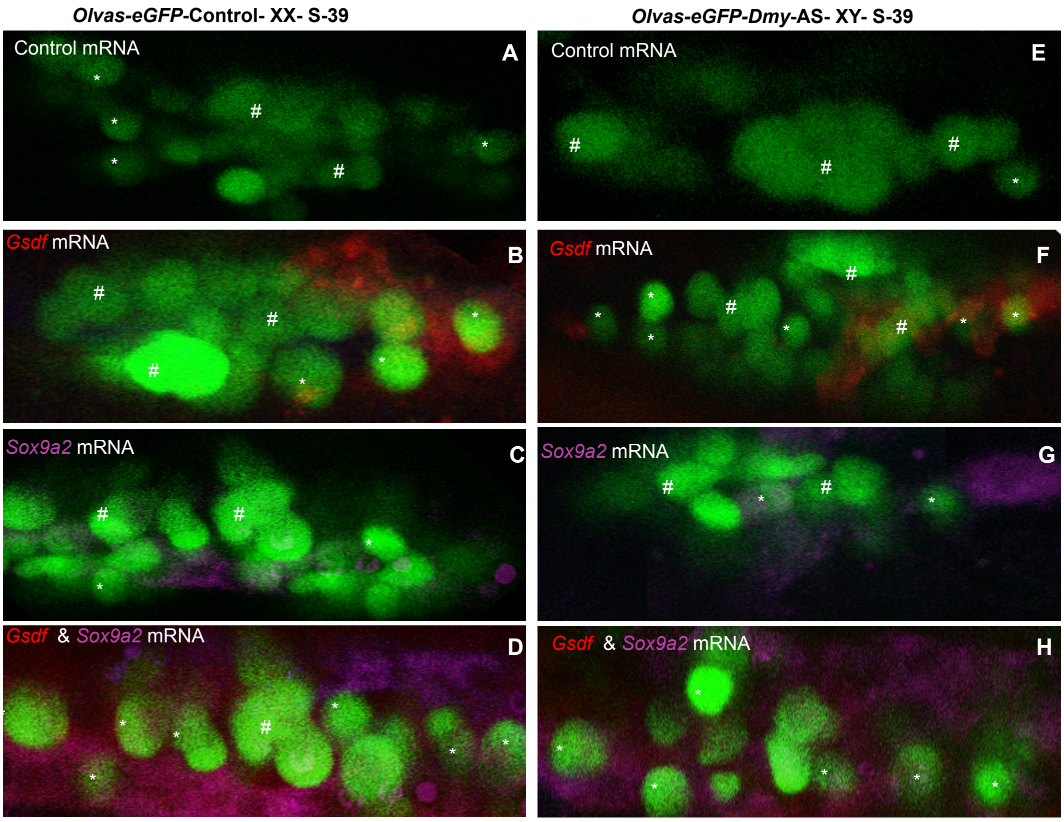

Supplementary figure 7. Schematics of breeding and mating strategies of Dmy-KD transgenic line. The adult individuals were analysed for genome integration and genetic sex before mating in each generation.

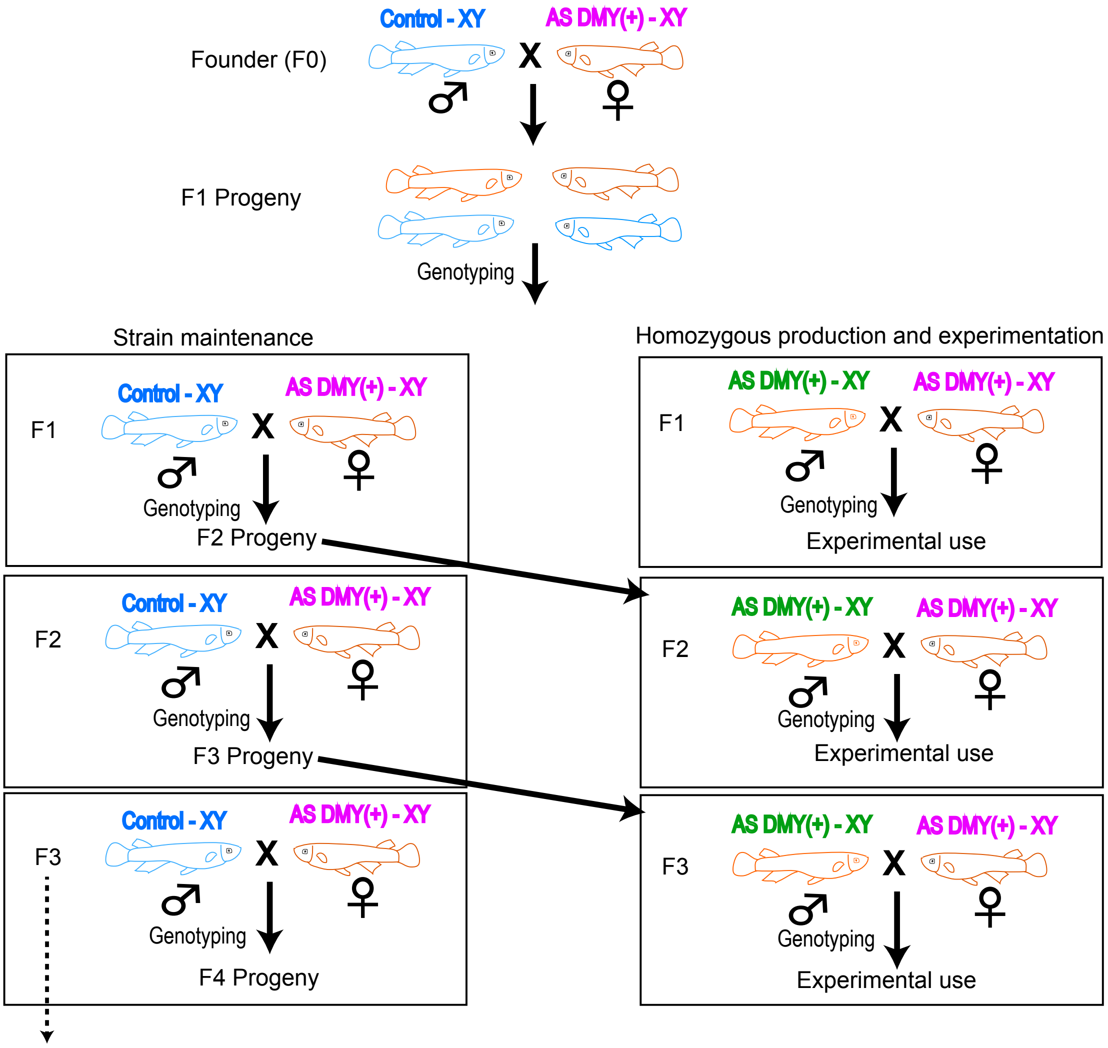

Supplement: Supplementary Information [file srep19480-s1.pdf]
